# Experimenter-free pain assessment in mice using a thermal gradient ring and functional linear models

**DOI:** 10.1097/PR9.0000000000001469

**Published:** 2026-07-10

**Authors:** Aketzali Garcia, Justin N. Siemian, Gabriel Loewinger, Prerna M. Yadav, Sarah Sarsfield, Francisco Pereira, Yeka Aponte

**Affiliations:** a Neuronal Circuits and Behavior Section, National Institute on Drug Abuse Intramural Research Program, National Institutes of Health, Baltimore, MD, USA; b Machine Learning Team, Section on Functional Imaging Methods, National Institute of Mental Health, Bethesda, MD, USA; c The Solomon H. Snyder Department of Neuroscience, Johns Hopkins University School of Medicine, Baltimore, MD, USA

**Keywords:** Thermal gradient ring, Functional linear models, Thermal preference, Locomotor side effects, Pharmacotherapies, TRV734

## Abstract

**Introduction:**

Traditional preclinical laboratory animal tests of pain can be suboptimal for many reasons, such as experimenter bias, interference of motor effects, and a lack of emotional or motivational pain process engagement. These shortcomings may also impede the search for novel pain therapies. Recently, thermal preference or gradient tests have gained popularity, and several apparatuses are either commercially available or can be self-made. The thermal gradient ring (TGR) offers a high-resolution thermal preference assessment and removes spatial confounds that may arise in two-chamber or linear designs.

**Objectives:**

The aim of this study was to determine the effectiveness of the TGR as a tool to assess pharmacotherapies and develop an appropriate statistical framework for data analysis.

**Methods:**

We examined the effects of 17 treatment conditions, including pronociceptive and antinociceptive compounds, in male and female mice. Behavioral data were analyzed using functional linear models (FLM), enabling hypothesis testing of temperature preference and motor activity at each timepoint of the TGR session.

**Results:**

Functional linear models captured the temporal dynamics and magnitudes of treatment effects and identified locomotor side effects that were obscured by traditional session-averaged analyses. Minute-by-minute comparisons revealed distinct temporal progressions of treatment effects and locomotor side effects.

**Conclusion:**

Our findings demonstrate that the TGR-FLM combination is a robust framework and a valuable tool for screening novel pain therapies.

## Abstract 2

Supplemental Digital Content is Available in the Text.

Combining a thermal gradient ring and functional linear models allows high-resolution screening of temperature preference and locomotor activity for pronociceptive and antinociceptive treatments.

## 1. Introduction

Chronic pain is a public health challenge with few novel therapies emerging over the past 60 years.^18,33^ One reason for the lack of breakthrough medicines may be the reliance of preclinical studies on experimenter-judged measurements of behavioral responses in acute pain tests.^38,45^ One such assay, the hot plate test, measures heat-evoked nocifensive behaviors. This assay offers face validity and simplicity, but nociceptive latency measurements are typically performed by experimenters, introducing potential bias based on experimental expectations. Moreover, reflexive responses rely on intact motor coordination,^8,26^ making it difficult to distinguish analgesic effects from sedation or motor impairment. Therefore, supplementary assessments (eg, open field) are often necessary.^32^

Two-zone and continuous temperature gradient tracks assess behavior at nonextreme temperatures that better reflect thermal allodynia in chronic pain patients.^1,4,5^ Two-zone paradigms minimize handling, enable bias-free data collection, and permit unrestricted locomotion between zones, but choosing optimal temperature pairs can be challenging.^1,26^ Continuous linear gradients assess temperature sensitivity across a broad range^6,29^ but introduce spatial confounds. Rodents demonstrate thigmotaxis, and the thermoneutral zone lies in the most-exposed middle of the track. These competing spatial and thermal preferences may delay preferred thermal zone selection or make tests susceptible to anxiolytic rather than antinociceptive effects.

To address these limitations, circular tracks known as thermal gradient rings (TGRs) were developed to provide spatial uniformity. Symmetrical gradients allow data point duplication and increased sensitivity to locomotor effects because animals are not required to turn around.^42,43^ The TGR has been used to assess thermal sensitivity in mice lacking TRPM8, TRPA1,^42,47^ and TRPV3 channels^21^ as well as in neuropathic pain,^39^ thermogenesis,^7^ and heroin withdrawal studies.^14^ Given this ability to assess temperature sensitivity and locomotion simultaneously, we propose that the TGR is a valuable tool for in vivo screening of analgesics.

Here, we use a TGR to detect changes in thermal sensitivity and locomotion for various pronociceptive and antinociceptive treatments, including emerging therapeutic candidates. To analyze behavioral dynamics, we used functional linear models (FLM), enabling hypothesis testing at each timepoint. Functional linear models overcome the limitations of session-averaged statistics, which may obscure meaningful time-dependent effects. For example, 2 compounds may seem equally effective when evaluated by session-averaged temperature preference, although they differ in magnitude and action duration. Drug A might produce a strong but short effect, while Drug B produces weaker but sustained changes; averaging responses across the session would falsely suggest comparability. Similarly, a compound with bidirectional effects (eg, early pronociception followed by later antinociception) might seem to have no effect. Using FLM, we compared, minute-by-minute, the magnitudes and temporal progressions of treatment effects and locomotor side effects. Our findings highlight the utility of the TGR and FLM as a combined framework for characterizing novel pain therapeutics.

## 2. Methods

### 2.1. Animals

Two- to six-month-old male and female wildtype mice (C57BL/6J background) weighing 17 g to 35 g were used. Mice were randomly assigned to groups while accounting for sex-matched and age-matched controls. Mice were group-housed with littermates on a 12-hour light/dark cycle with ad libitum access to water and rodent chow. All experimental protocols were conducted in accordance with the US National Institutes of Health Guidelines for the Care and Use of Laboratory Animals and with the approval of the National Institute on Drug Abuse Animal Care and Use Committee.

### 2.2. Behavioral assay

The TGR (Cat # 35550, Ugo Basile SRL, Gemonio, Italy) is a circular track previously described.^42^ Ambient room temperature during testing was 22 ± 1°C, verified by a temperature probe in the room, and apparatus temperature was confirmed with a calibrated infrared temperature gun at session beginning and end. Before testing, mice were placed on the TGR for a 30-min habituation session with both temperature controllers set to 22°C. Habituation occurred 1 to 5 days before testing. For testing, temperature controllers were set to 5°C and 52°C and 12 zones were established with the same temperatures occurring on both sides of the ring. Individual temperature zones covered surface areas of ≈23 cm^2^ with an increment of 4.3°C between zones.

Mice were treated with one experimental condition and placed on the 22°C zone. Experimental conditions are detailed in Supplemental Materials. After setting the mouse in the apparatus, the experimenter left the room. Tests proceeded for 1 hour for all treatment conditions except for the TRV734 experiment, which was 2 hours.

Sessions were recorded with ANY-maze v6 software (RRID:SCR_014289; Stoelting Co, IL), which tracked the center point of the mouse. The raw tracking data exported were zone occupancy, zone entries, and speed (m·s^−1^). To assess drug-related locomotor activity, we used speed rather than total distance, as speed reflects active locomotion and minimizes inflation from oscillatory movements.^41^

Temperature preference was calculated using the following formula.^42^

$$Temperature\;Preference=\frac{\left[\left(Time\;spent\;at\;5{}^{\circ}C*5{}^{\circ}C\right)+\left(Time\;spent\;at\;9.3{}^{\circ}C*9.3{}^{\circ}C\right)+\ldots \;\left(Time\;spent\;at\;52{}^{\circ}C*52{}^{\circ}C\right)\right]\;}{Time\;spent\;at\;5{}^{\circ}C+Time\;spent\;at\;9.3{}^{\circ}C+\ldots \;Time\;spent\;at\;52\;{}^{\circ}C}$$

Temperature preference was calculated in 1-min bins and averaged for 5-, 10-, and 60-min periods.

### 2.3. Quantification and statistical analysis

GraphPad Prism v10 (RRID:SCR_002798; GraphPad Software, CA) and MATLAB R2023b (RRID:SCR_001622; MathWorks, MA) were used for graphing and data visualization, except for FLM graphs. A formal power analysis was not conducted to predetermine sample size; group sizes were selected based on prior TGR studies.^14,21,39,42,43^ Pearson correlation, unpaired Student's *t*-tests, one-way and 2-way repeated-measures (RM) ANOVAs, mixed-effects RM, and Dunnett or Šidák corrections for multiple comparisons were performed. Data are plotted as mean ± s.e.m. unless otherwise noted in the figure legend, and statistical values (*F*, *t*, and *P*) are noted in figure legends.

FLM were fit with the *fosr()* function^20,35^ and *pffr() function*^40^ in the R package *refund*.^13^ Results in the first set of models come from 2 separate functional ANOVAs: one with temperature preference as the functional outcome, and the other with locomotor speed. Each functional ANOVA model estimates the mean difference in temperature preferences of animals in a given treatment group relative to the baseline treatment group (saline) at each individual timepoint. Multiple comparisons across the 60-minute session were corrected with the Benjamini−Hochberg (BH) procedure^2^ and significant timepoints are indicated by blue-shaded confidence interval bands in the functional coefficient plots. The *concurrent* FLM model is akin to a concurrent functional ANCOVA. In addition to the treatment group factor variable, this model included linear and quadratic terms for average locomotion speed at each session timepoint (ie, as functional covariates). This analysis was used to compare mean temperature preferences between groups, while adjusting for a linear effect of locomotor activity (speed) at each time bin. For each model, we checked for heteroscedasticity in the outcome across treatment groups by examining the distribution of the squared residuals pooled across timepoints *s*. For any models that showed signs of heteroscedasticity, we fit a generalized least squares functional regression (GLS) with the *gls_cs()* function in the refund package.^12^ Some GLS models failed to fit due to singularity of the working covariance matrix. In these cases, we estimated functional coefficient estimate variance with a nonparametric bootstrap with 1000 replicates to ensure valid inference for the coefficient estimates. A detailed description of FLM analysis is in Supplemental Materials.

### 2.4. Data availability

Data are available on Open Science Framework (OSF), https://doi.org/10.17605/OSF.IO/7CWD6.

## 3. Results

### 3.1. The thermal gradient ring robustly detects changes in temperature preference and locomotor activity

**Figure 1.**
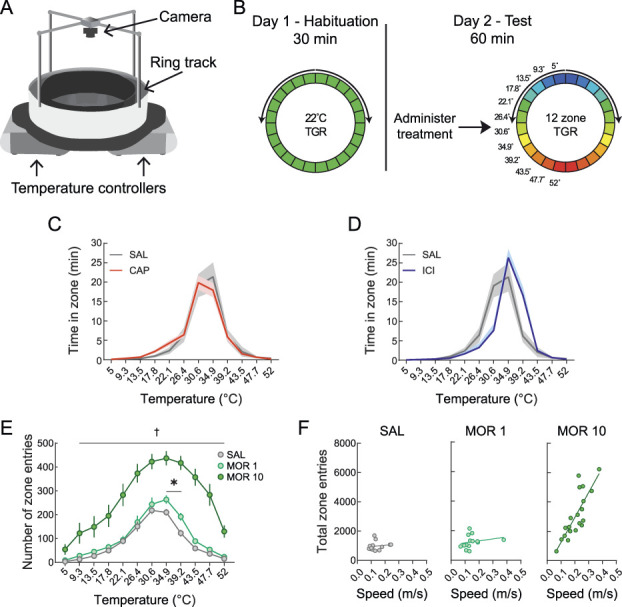
The TGR identifies changes in temperature preference and locomotor activity across sessions. (A) Ugo Basile TGR schematic. An aluminum ring track sits above 2 temperature controllers, and a camera is positioned above for behavior tracking. (B) On day 1, both temperature controllers were set to 22°C and mice were habituated to the apparatus for 30 minutes. On day 2, the TGR temperature controllers were set to 5°C and 52°C to create a gradient of 12 zones per side. After treatment administration, mice were placed in the middle of the temperature gradient (∼26.4°C temperature zone) and allowed to freely move for 60 minutes. (C) Zone occupancy distribution after saline (SAL) and capsaicin (CAP) administration. (D) Zone occupancy distribution after SAL and icilin (ICI) administration. (E) Morphine administration significantly increases the number of entries per zone relative to saline. Two-way RM ANOVA: zone temperature × treatment interaction *F* (22, 495) = 6.261, *P* < 0.0001, Dunnett post-test: MOR 1 vs SAL (*P < 0.05), MOR 10 vs SAL (^†^*P* < 0.05). MOR 1, 1 mg/kg; MOR 10, 10 mg/kg (i.p.). (F) Relationship between total zone entries and speed after SAL (left; Pearson correlation: *R*^2^ = 0.02, *P* = 0.5949), MOR 1 (middle; Pearson correlation: *R*^2^ = 0.05, *P* = 0.4372), and MOR 10 administration (right; Pearson correlation: *R*^2^ = 0.66, *P* < 0.0001).

We used the TGR (Fig. 1A) as an unbiased, experimenter-free assay to detect acute changes in thermal sensitivity and locomotor side effects induced by well-known analgesic, pronociceptive, and non–pain-indicated treatments (Table 1). For all conditions, TGR temperature gradients were set from 5 to 52°C in a room with an ambient temperature of 22°C (Fig. 1B). Saline (SAL) was administered as a control through intraplantar (i.pl.) or intraperitoneal (i.p.) injection. Both routes elicited comparable temporal dynamics of temperature preference and locomotor activity with no significant differences observed between injection groups (see supplemental digital content, Fig. S1, http://links.lww.com/PR9/A420).

**Table 1:** Treatments and expected TGR results.

Treatment	Pharmacology	Effect/clinical use	Expected effect on thermal preference	Expected effect on locomotion
2-BFI	Imidazoline I2 receptor agonist	In preclinical studies, produces antinociception and hypothermia	Warmer	Decrease
Cannabidiol	Mixed pharmacology at cannabinoid receptor 1 and 2	FDA-approved epilepsy treatment, non-FDA approved for many other conditions including pain	None	None
Capsaicin	Transient receptor potential cation channel subfamily V member 1 agonist	Activates heat receptors to produce warming or burning sensation, used in topical formulations for analgesia	Colder	None
Cocaine	Monoamine reuptake inhibitor, increases synaptic concentration of dopamine, norepinephrine, and serotonin	Psychostimulant effects	None	Increase
Complete Freund's adjuvant (1 d and 21 d)	Induces local inflammatory response	N/A	Colder	None
Duloxetine	Serotonin–norepinephrine reuptake inhibitor	Used to treat depression and chronic pain	Warmer	None
Gabapentin	Ligand of α_2_δ subunit of voltage-gated calcium channels	Analgesic for neuropathic pain	Warmer	Decrease
Haloperidol	Antagonist at D2-like dopamine receptors and 5-HT_2A_ and 5-HT_2C_ receptors, many others	Treatment for schizophrenia and related disorders. Induces hypothermic effect in rodents	Warmer	Decrease
Icilin	Transient receptor potential M8 agonist	Evokes cold-like sensations similar to menthol	Warmer	None
Morphine (1 and 10 mg/kg)	µ-opioid receptor agonist	Inhibits nociceptive transmission, also affects mesolimbic dopamine system	Broader	Increase
Morphine-induced hyperalgesia	See morphine	Preclinical and clinical phenomenon of increased pain sensitivity after discontinuation of opioids	Narrower	None
TRV734 (1, 3, 10, and 30 mg/kg)	G-protein biased ligand of the µ-opioid receptor	Compound in development for the treatment of opioid use disorder and pain management	Broader	Increase
Saline	Control	N/A	N/A	N/A
2-Hydroxypropyl–β- cyclodextrin	Control	N/A	N/A	N/A

Previous studies showed that a TGR detects abnormal thermal behavior in mice lacking thermosensitive transient receptor potential (TRP) channels.^21,39,42,43^ Therefore, as proof of concept, we evaluated changes in thermal sensitivity using TRP agonists. We injected the TRPV1 agonist capsaicin (CAP) or the TRPM8 agonist icilin (ICI) into the hind paw of mice. Assessment of the time spent in each zone showed that the SAL control group spent more time in the 30.6–34.9°C range over a 60-minute session, while CAP and ICI groups exhibited peak temperatures of 30.6°C and 34.9°C, respectively (Fig. 1C, D). Given the transient nature of capsaicin-induced nociception, we examined temperature preference using 5- and 10-minute time blocks (see Fig. S2, supplemental digital content, http://links.lww.com/PR9/A420). We found that CAP induced a significant preference for colder zones during the first 5 minutes (see Fig. S2A, supplemental digital content, http://links.lww.com/PR9/A420), with an average temperature of 29.26 ± 1.22°C vs SAL at 34.45 ± 1.26°C.

To evaluate the ability of the TGR to detect drug-induced motor effects, we next examined morphine, an opioid with known locomotor side effects.^15^ Mice were injected with morphine at doses of 1 or 10 mg/kg (i.p.; MOR 1 and MOR 10, respectively) and were placed on the TGR 30-minutes postinjection. The MOR 10 group significantly increased the number of zone entries compared with the SAL group (Fig. 1E). Notably, zone entries and speed were positively correlated, a relationship absent in SAL and MOR 1 (Fig. 1F), indicating that the number of zone entries is a good indicator of locomotor activity.

We subsequently tested treatments previously reported as pronociceptive, analgesic, or non–pain-indicated (Table 1).^10,11,17,19,22,25,30,31,37^ We expected pronociceptive compounds to bias animals toward cooler temperatures and antinociceptive treatments toward warmer zones (Table 1). Pronociceptive manipulations included agents that induce inflammatory pain (eg, complete Freund's adjuvant [CFA]) and nociceptive sensitization caused by chronic opioid exposure (eg, morphine-induced hyperalgesia [MIH]). Analgesic (or antinociceptive) compounds included non-opioids (eg, gabapentin [GAB], used to treat nerve pain) and opioids. Non–pain-indicated treatments included cocaine (COC, psychostimulant), haloperidol (HAL, antipsychotic), duloxetine (DLX, antidepressant), and cannabidiol (CBD). We first examined session-averaged zone occupancy and zone entries for each compound relative to SAL (see Fig. S3, supplemental digital content, http://links.lww.com/PR9/A420). Pronociceptive manipulations did not produce sustained shifts in zone occupancy at the session level. By contrast, some antinociceptive treatments appeared to be associated with warmer temperature zone occupancy (eg, GAB; see Fig. S3I, supplemental digital content, http://links.lww.com/PR9/A420). Similarly, DLX, a drug with secondary analgesic effects (see Fig. S3J, supplemental digital content, http://links.lww.com/PR9/A420) shifted preference to warmer temperatures. As expected, MOR 10 and COC administration broadened the range of zones visited and reduced the time spent in neutral temperatures (see Fig. S3G and M, supplemental digital content, http://links.lww.com/PR9/A420), consistent with increased locomotor activity.

### 3.2. Functional linear models analysis reveals pharmacobehavioral temporal dynamics

**Figure 2.**
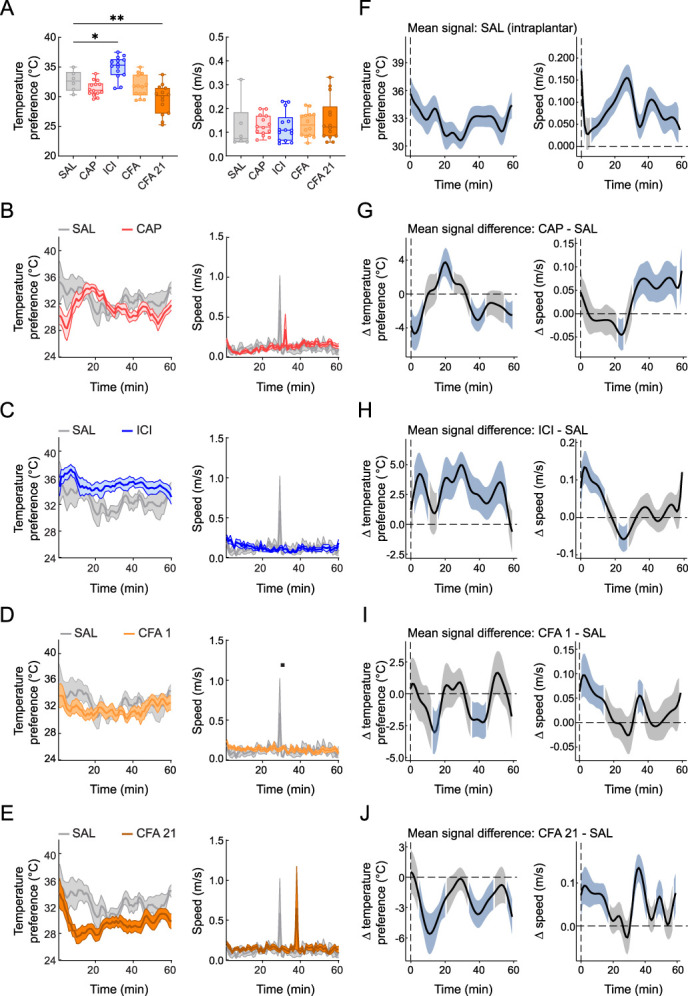
Functional linear models identify significant behavioral effects induced by TRP agonists and proinflammatory treatments beyond those detected by traditional analyses. (A) Session-averaged temperature preference and speed over a 60-minute session after intraplantar administration of saline (SAL), capsaicin (CAP), icilin (ICI), acute complete Freund's adjuvant (CFA 1), and chronic CFA (CFA 21). One-way ANOVA, Dunnett post-test **P < 0.01, *P < 0.05. Box plots show the median. (B−D) Time-course plots of mean temperature preference (*left*) and speed (*right*) for CAP, ICI, and CFA 21 compared with SAL over the full session. No significant treatment × time interactions were detected (2-way RM ANOVA for temperature preference; mixed-effects RM for speed). Solid lines indicate the mean across mice at each timepoint, and shaded areas represent the pointwise s.e.m. calculated across animals. These plots provide a descriptive visualization of average trajectories and between-animal variability. (E) SAL group mean signals for temperature preference and speed. See supplemental digital content (Table S1, http://links.lww.com/PR9/A420) for detailed statistics. (F–H) FLM analysis coefficient estimates of the difference in average temperature (*left*) and speed (*right*) for each compound vs the control group (SAL). Unlike session-average analysis, FLM reveals that (G) CAP and (H) ICI administration lead to statistically significant preferences for cooler and warmer temperatures at different temporal windows, respectively. Notably, CAP induces a preference for warmer temperatures for a short time interval and then returns to cold preference. (I−J) Inflammatory pain induced by CFA 1 (acute) and CFA 21 (chronic) treatment promotes a significant preference for cooler zones and increases speed in different time windows. Blue shading indicates timepoints with statistically significant effects after Benjamini−Hochberg correction (ie, where the mean significantly differs from zero).

Session-averaged temperature preference and speed were first calculated for intraplantar treatments, including CAP, ICI, CFA 1 (acute), and CFA 21 (chronic) (Fig. 2A). Using these traditional summaries, we observed increased temperature preference after ICI administration and decreased preference after CFA 21, whereas no sustained effects were detected for CAP or CFA 1. Session-averaged speed did not differ significantly across treatments. As previously reported, averaging data across an entire 60-minute session can underperform relative to more segmented behavioral analyses.^46,48^ Therefore, we next evaluated average minute-by-minute temperature preference and motor activity induced by each treatment (Fig. 2B–E). However, this approach does not allow hypothesis testing of differences across treatments at each session timepoint. We thus implemented FLM to evaluate how treatment effects evolve over time. Previous work has demonstrated the advantages of using functional linear mixed models for trial-by-trial temporal analysis in fiber photometry experiments.^23^ The present analyses do not require random effects, as we only observed a single functional outcome (ie, a single session) in this experiment.

For the first analysis, we evaluated the changes in temperature preference and speed across time for the same treatments. Each treatment was compared with the control group (Fig. 2F–J). To assess heteroscedasticity, we examined the distribution of squared residuals pooled across time (see Fig. S4, supplemental digital content, http://links.lww.com/PR9/A420) and fit functional GLS models to account for heteroscedasticity. Unlike standard statistical tests on binned data (Fig. 2A–E, see Fig. S3, supplemental digital content, http://links.lww.com/PR9/A420), FLM demonstrated that CAP treatment yielded a statistically significant negative change followed by a positive change in temperature preference and then returned to negative preference later in the session compared with the SAL group (ie, preference for cooler temperatures; Fig. 2G). Moreover, CAP-treated mice also showed reduced speed at ∼20 minutes, followed by increased speed during the final 30 minutes. By contrast, ICI treatment led to a warmer temperature preference compared with SAL across nearly the entire session (Fig. 2H). Both CFA 1 and CFA 21 treatments significantly reduced mean temperature preference, accompanied by periods of increased speed when mice shifted toward warmer zones (Fig. 2I, J).

**Figure 3.**
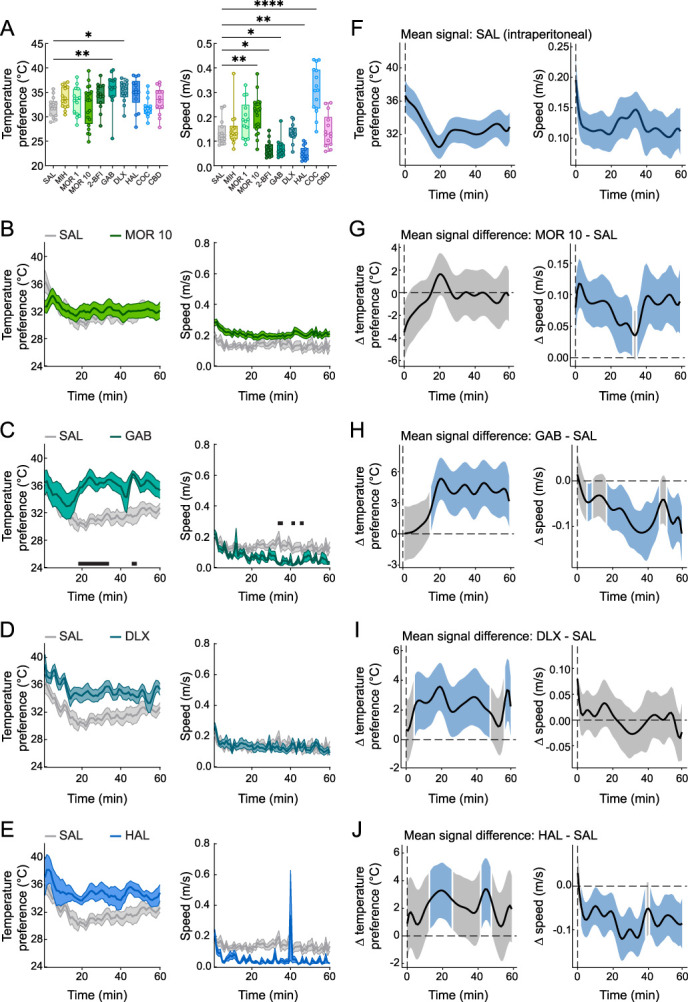
Functional linear models identify significant behavioral effects induced by antinociceptive and pronociceptive treatments and track their temporal dynamics. (A) Session-averaged temperature preference and speed for intraperitoneally administered treatments: morphine-induced hyperalgesia (MIH), 1 mg/kg morphine (MOR 1), 10 mg/kg morphine (MOR 10), 2-BFI I_2_R ligand (2-BFI), gabapentin (GAB), duloxetine (DLX), haloperidol (HAL), cocaine (COC), and cannabidiol (CBD). One-way ANOVA, Dunnett post-test, ****P < 0.0001, **P < 0.01, *P < 0.05. Box plots show the median. (B−E) Time-course plots of mean temperature preference (*left*) and speed (*right*) for MOR 10, GAB, DLX, and HAL vs SAL over 60 minutes. Only (C) GAB shows discrete timepoint changes in temperature preference and speed, indicated by black squares. Two-way RM ANOVA (temperature preference) and mixed-effects RM (speed), Šidák post-test (*P* < 0.05). Solid lines indicate the mean and shaded areas represent the pointwise s.e.m. calculated across animals. See supplemental digital content (Table S1, http://links.lww.com/PR9/A420) for detailed statistics. (F) SAL group mean signals for temperature preference and speed for intraperitoneal administration. (G–J) FLM analysis coefficient estimates of the difference in average temperature (left) and speed (right) vs the control group (SAL). FLM identifies treatment-specific temporal patterns not captured by traditional analyses. For example, (G) MOR 10 shows a significant increase in speed across most of the session, whereas (H) GAB, (I) DLX, and (J) HAL induce preference for warmer temperatures, although only GAB and HAL significantly decrease speed at different temporal windows. Blue shading indicates timepoints with statistically significant effects after Benjamini−Hochberg correction.

We next compared traditional analysis with FLM for compounds administered intraperitoneally (i.p.), each relative to its SAL group (Fig. 3 and Fig. S5, supplemental digital content, http://links.lww.com/PR9/A420). Session-averaged temperature preference and speed showed a preference for warmer temperatures accompanied by reduced locomotor activity for GAB and DLX, whereas HAL did not alter temperature preference but significantly decreased speed (Fig. 3A). As observed previously, MOR 10 increased locomotor activity, and COC produced a similar effect. Time-course analyses (Fig. 3B–E and see Fig. S5A−E, supplemental digital content, http://links.lww.com/PR9/A420) did not reveal sustained effects over the session, with the exception of GAB-treated mice, which showed transient increases in temperature preference at discrete timepoints (Fig. 3C), making it difficult to determine whether these effects were brief or sustained.

By contrast, FLM analysis (Fig. 3F–J) revealed temporal dynamics and magnitude effects that were not captured by traditional approaches. Using FLM, MOR 10 administration did not show significant changes in temperature preference but significantly increased locomotor activity (Fig. 3G). We also found that GAB, DLX, and HAL treatments led to positive increases in average temperature preference and decreases in motor activity with different temporal dynamics (Fig. 3H–J). By contrast, mice undergoing MIH showed no significant changes in temperature preference or speed (see Fig. S5F, supplemental digital content, http://links.lww.com/PR9/A420), and MOR 1 failed to affect temperature preference while increasing locomotor activity in the first minutes of the session (see Fig. S5G, supplemental digital content, http://links.lww.com/PR9/A420). Mice treated with imidazoline I2 receptor agonist (2-BFI) did not alter temperature preference but decreased locomotor activity at distinct timepoints during the session (see Fig. S5H, supplemental digital content, http://links.lww.com/PR9/A420). As expected, the COC group showed an increase in speed followed by deceleration; however, this treatment did not induce changes in temperature preference (see Fig. S5I, supplemental digital content, http://links.lww.com/PR9/A420). Finally, mice treated with CBD did not display significant changes in temperature preference but did exhibit a brief increase in mean speed during the first ∼20 minutes of the session (see Fig. S5J, supplemental digital content, http://links.lww.com/PR9/A420).

One explanation for the observed differences in temperature preference is that the compounds alter locomotor activity rather than temperature sensitivity. To explore this, we fit a concurrent model comparing temperature preference across treatment groups while adjusting for the effect of speed at each session timepoint. We included both linear and quadratic terms for speed to account, at least in part, for potential nonlinearity in the speed–preference relationship. Because GLS is not implemented, to the best of our knowledge, in the packages that fit concurrent models, we used a nonparametric bootstrap to generate 95% confidence intervals that should remain valid even in the presence of heteroscedasticity. Regression coefficients are interpreted as differences in temperature preference between groups after statistically adjusting for locomotor speed (with linear/quadratic terms).

We first assessed whether the lack of temperature preference change observed in both MOR 10 and COC groups (Figs. 3G and see Fig. S5I, supplemental digital content, http://links.lww.com/PR9/A420) was due to drug effects on locomotion. After adjusting for speed, neither MOR 10 nor COC induced changes in temperature preference (see Fig. S6A and B, supplemental digital content, http://links.lww.com/PR9/A420). This suggests that increased locomotion is not associated with the lack of temperature preference in either group. Subsequently, GAB and HAL were selected to determine whether significant temperature preference changes originally observed were due to drug-induced decreases in speed (Fig. 3H and J). After adjusting for speed, neither drug revealed significant changes compared with SAL (see Fig. S6C and D, supplemental digital content, http://links.lww.com/PR9/A420), suggesting that the preference for warmer temperatures previously observed may be mediated through decreased speed. Together, these results indicate that the FLM statistical framework can reliably detect drug temporal dynamics and effect magnitude in behavioral TGR time series data that are obscured by traditional summary analyses.

### 3.3. Using the thermal gradient ring and functional linear models to evaluate novel pain therapies

Finally, we applied the TGR-FLM framework to characterize a novel therapeutic compound. TRV734 is an oral G-protein ligand that selectively binds to the µ-opioid receptor with high affinity. In nonclinical studies, TRV734 has demonstrated a potential analgesic effect with decreased gastrointestinal motility compared with morphine at equianalgesic doses.^16,36^ Furthermore, a recent study in humans has shown that the analgesic effect of TRV734 during a cold pain test was similar to oxycodone.^36^ Therefore, we examined the effects of acute doses of TRV734 (1, 3, 10, and 30 mg/kg, i.p.) using the TGR.

**Figure 4.**
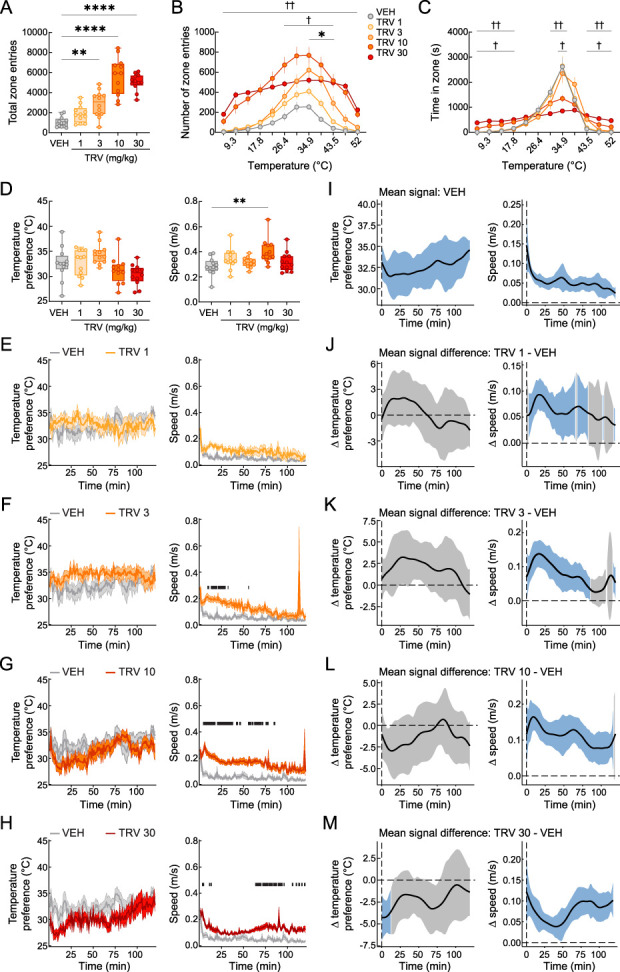
Temporal dynamics of acute TRV734 administration in temperature preference and speed. (A) Average total number of zone entries over a 2-hour session. TRV734 (TRV) administration significantly increases the number of zone entries compared with vehicle (VEH). One-way ANOVA with Dunnett post-test, **P < 0.01, ****P < 0.0001. Box plots show the median. (B) Average number of zone entries per temperature zone. Higher doses (TRV 10 and 30) significantly increase zone entries across all temperature zones relative to vehicle (VEH). Two-way RM ANOVA, zone entries × dose interaction *F* (44, 605) = 5.734, *P* < 0.0001, Dunnett post-test: TRV 1 vs SAL (*P < 0.05), TRV 3 vs SAL (†*P* < 0.05), TRV 10 and TRV 30 vs SAL (††*P* < 0.05). (C) Average time spent per temperature zone. TRV 10 and 30 increase occupancy of extreme temperatures and reduce time spent in neutral zones compared with VEH. Two-way RM ANOVA, zone entries × dose interaction *F* (44, 605) = 4.930, *P* < 0.0001, Dunnett post-test: TRV 10 vs SAL (†*P* < 0.05), TRV 30 vs SAL (††*P* < 0.05). (D) Session-averaged temperature preference and speed summary. One-way ANOVA, Dunnett post-test, **P < 0.01. (E−H) Time-course plots of mean temperature preference (*right*) and speed (*left*) for TRV 1, 3, 10, and 30 mg/kg compared with VEH. These analyses do not reveal sustained changes in temperature preference but show increased locomotor speed at discrete timepoints after TRV administration at 3, 10, and 30 mg/kg. Black squares denote statistically significant timepoints. Two-way RM ANOVA (temperature preference) and mixed-effects RM (speed), Šidák post-test (*P* < 0.05). Solid lines indicate the mean and shaded areas represent the pointwise s.e.m. calculated across animals. See supplemental digital content (Table S1, http://links.lww.com/PR9/A420) for detailed statistics. (I) Temperature preference and speed mean signal for the vehicle (VEH) group. (J–M) FLM coefficient estimates of the difference in the average temperature (left) and speed (right) for TRV 1 and 10 vs VEH. (J) TRV 1 and (K) TRV 3 show statistically significant increases in locomotor activity in the ∼0–75-minute time interval compared with VEH. (L) TRV 10 and (M) TRV 30 promote locomotor activity across the entire session. TRV 30 promotes preference for cooler zones during the first 20 minutes of the session. Blue shading indicates timepoints with statistically significant effects after Benjamini−Hochberg correction.

Session-averaged analyses showed increased total zone entries at 3, 10, and 30 mg/kg relative to vehicle (VEH) (Fig. 4A), with higher doses broadening zone occupancy and increasing the number of zones visited (Fig. 4B, C). Session-averaged temperature preference remained unchanged relative to VEH (Fig. 4D, left), and session-averaged speed differed from VEH only at 10 mg/kg (Fig. 4D, right). No sex-dependent effects were observed for either temperature preference or locomotor activity (see Fig. S7, supplemental digital content, http://links.lww.com/PR9/A420). Time-course analyses suggested changes in speed for all doses except 1 mg/kg (Fig. 4E–H).

FLM analysis was then applied to explore the temporal dynamics of TRV734 effects. Functional GLS could not be implemented because it yielded noninvertible working covariance matrices. Therefore, we used a nonparametric bootstrap to estimate coefficient variance and construct 95% confidence intervals. We found that lower doses (1 and 3 mg/kg) led to an increase in speed during the ∼0–75 minute interval relative to the VEH group (Fig. 4I–K). Moreover, we observed that higher TRV734 doses (10 and 30 mg/kg) increased locomotor activity throughout the session (Fig. 4L and M). In addition, significant preference for cooler zones was detected with the highest dose (Fig. 4M).

## 4. Discussion

Historically, behavioral tests have been designed with a limited number of dependent measures that could be easily collected and analyzed through statistical testing to draw a relative conclusion. Statements to the effect of “*treatment X affected behavior A but not behavior B*″ are technically valid, but this design may obscure other potentially important behavioral changes. For example, placing an animal in a conditioning chamber and collecting only lever-press data may miss changes in other behaviors (eg, locomotion, grooming, stereotypy) that could better describe the overall impact of treatment. For pain research, measuring sensory pain behavior in situations where emotional/motivational pain, motor effects, or other subtle changes cannot be simultaneously monitored has perhaps prolonged the search for novel pain therapeutics.

In this context, we used a TGR apparatus^42^ optimized to detect changes in thermal preference and locomotor behavior in mice after administration of nociceptive or antinociceptive compounds. Although thermal preference is not a common clinical symptom of chronic pain, it remains a robust, mechanistically interpretable outcome in preclinical models. Unlike classic acute pain tests, such as the hot plate, which only measure acute withdrawal latencies, the TGR captures multiple behaviors simultaneously and within a single session. The single-session format minimizes effects from learning, habituation, or cumulative drug exposure, although it does not capture longer-term processes such as adaptation, sensitization, or tolerance. Our primary aim was to assess the utility of the TGR as a screening tool for pronociceptive and antinociceptive treatments. Unlike previous studies,^42,43^ we used an extended gradient to provide access to more extreme temperatures, increasing sensitivity to robust antinociceptive effects while enabling detection of potential locomotor side effects. However, mice may avoid very cold or hot zones regardless of treatment, which could limit interpretation of extreme-zone occupancy.

Our work highlights the importance of using time-resolved statistical analyses. Traditional analyses of session averages can obscure effects. For example, FLM revealed significant effects that were masked when data were summarized in 60-minute bins or 1-minute intervals (eg, see CAP, CFA 1, MOR 1, and CBD analyses). Therefore, we propose the use of a TGR together with FLM as an experimental and analysis framework for evaluating pain models and pain treatments.

Functional linear models revealed effects obscured by standard methods, echoing similar findings when using functional regression methods to analyze neural data.^23^ Functional linear models provide a framework to compare the temporal dynamics of signals between treatment groups in both time and magnitude. Moreover, the resulting plots provide a simple and interpretable visualization: each graph illustrates the changes in magnitude for a decrease or increase in temperature or speed, the pointwise 95% CI for the entire time series, and the time window of statistical significance after BH correction.

Implementing TGR-FLM analysis revealed noticeable effects for several treatments. Proof-of-concept treatments capsaicin and icilin shifted mice toward colder and warmer temperatures, respectively, with different temporal dynamics. Drugs with known abuse-liability such as morphine and cocaine increased locomotor activity and zone exploration, likely due to their enhancement of mesolimbic dopamine transmission.^44^ However, these drugs did not have significant changes in magnitude of temperature preference over time. In addition, mice with morphine-induced hyperalgesia exhibited a trend toward warmer temperatures, suggesting that the TGR can be used to study pain sensitivity during opioid spontaneous withdrawal^14^ and to evaluate new therapies for opioid use disorder (OUD). In this context, we tested the dose-dependent effects of TRV734, an emerging medication for the treatment of OUD,^16,36^ on temperature preference and locomotor activity. Unlike morphine, the highest TRV734 dose resulted in a brief preference for cooler temperatures. Further studies in rodents will continue to elucidate the analgesic properties and potential side effects of this new treatment.

We also evaluated non-nociceptive compounds with secondary analgesic potential, including DLX and HAL, which exhibited effects similar to GAB, a non-opioid analgesic used to treat neuropathic pain. All 3 promoted a preference for warmer zones with GAB and HAL exhibiting decreased motor activity. While DLX is mainly prescribed for major depressive disorder, it is also used for chronic pain.^3,34^ Haloperidol, an antipsychotic, has been shown to enhance the antinociceptive effects of morphine^24^ but induces hypothermia as a side effect. Our findings suggest that these treatments may offer more clinically relevant options for pain management.

Future studies could apply FLM to zone-specific analyses to quantify the proportion of time spent in each zone over time, thereby dissecting treatment effects with greater resolution. In addition, we acknowledge sex as a significant biological determinant of nociception^9,27,28^; however, the limited sample size (∼3–7 mice per sex per treatment) rendered our FLMs underpowered for detecting sex-specific effects (data not shown). Increasing sample sizes for each sex is important for future studies. In addition, incorporating pose estimation methods such as DeepLabCut, SLEAP, or MoSeq may enhance the accuracy and objectivity of behavioral scoring in preclinical pain research. Integrating automated pose estimation for classical assays with experimenter-free methods such as the TGR and a robust statistical framework will be essential for advancement in the field.

In summary, a TGR makes it possible to assess nociception and analgesia in mice through temperature preference in an experimenter-free fashion. When combined with FLM, a TGR offers a robust framework for monitoring locomotor activity, detecting potential off-target effects evoked by pharmacological treatments, and analyzing temporal dynamics and effect magnitude. This experimental approach can be easily applied to novel treatments or pain models to identify those most suitable for further study.

## Disclosures

The authors have no conflict of interest to declare.

## Supplemental digital content

Supplemental digital content associated with this article can be found online at http://links.lww.com/PR9/A420.
