# Supplementary material for: Experimenter-free pain assessment in mice using a thermal gradient ring and functional linear models
Source: Pain Rep. 2026 Jul 10;11(4):e1469. doi: 10.1097/PR9.0000000000001469 (PMC13363194; doi:10.1097/PR9.0000000000001469)
Supplement: Supplementary file 2 [file painreports-11-e1469-s002.pdf]

Supplementary Materials for

Experimenter-free pain assessment in mice using a thermal gradient ring and functional linear models

Aketzali Garcia*, Justin N. Siemian*, Gabriel Loewinger*, Prerna M. Yadav, Sarah Sarsfield, Francisco Pereira, and Yeka Aponte[#]

*Contributed equally
[#] Corresponding author: yeka.aponte@nih.gov

- Figs. S1 – S7
- Supplementary Methods
    - Experimental Treatments
    - Functional Linear Models (FLM) Analysis
- References
- Table S1 (Statistical analysis details)

Figure S1

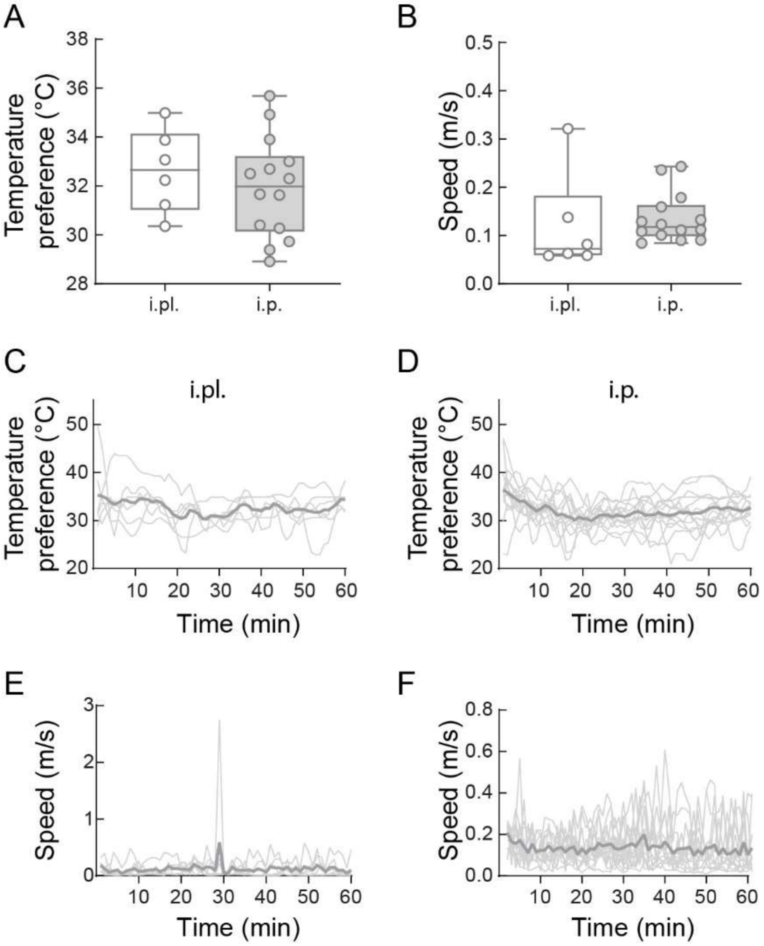

Fig. S1. Administration route does not elicit effects between saline-treated groups.

(A–B) Temperature preference and speed of mice administered saline via intraperitoneal (i.p.) and intraplantar (i.pl.) injection routes over a 60-min session. **(C–D)** Temperature preference and speed **(E−F)** over time after SAL i.p. and i.pl. administration. Dark lines represent group means; light lines represent individual subjects. See **Table S1** for detailed statistics.

Figure S2

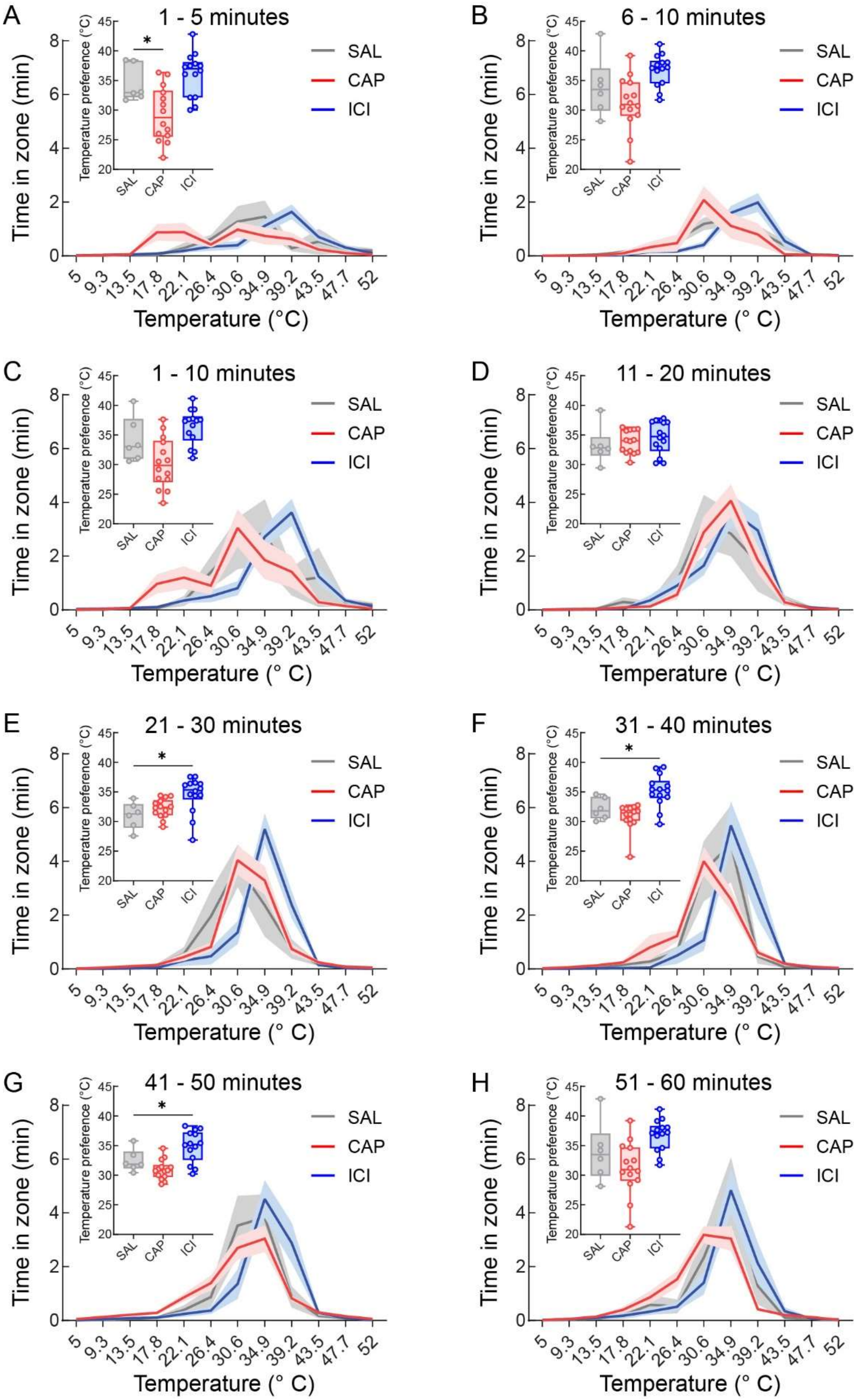
A
1 - 5 minutes
B
6 - 10 minutes
C
1 - 10 minutes
D
11 - 20 minutes
E
21 - 30 minutes
F
31 - 40 minutes
G
41 - 50 minutes
H
51 - 60 minutes
SAL
CAP
ICI
Time in zone (min)
Temperature (°C)
Temperature preference (°C)
5
9.3
13.5
17.8
22.1
26.4
30.6
34.9
39.2
43.5
47.7
52

Fig. S2. Changes in temperature preference over time after capsaicin and icilin administration. **(A–B)** Saline (SAL), capsaicin (CAP), and icilin (ICI) zone occupancy and temperature preference (inset) analyzed in 5-min intervals. **(C–H)**. Zone occupancy and temperature preference (inset) for the same groups analyzed in 10-min intervals. One-way ANOVA, Dunnett post-test, $^*P < 0.05$. See **Table S1** for detailed statistics.

Figure S3

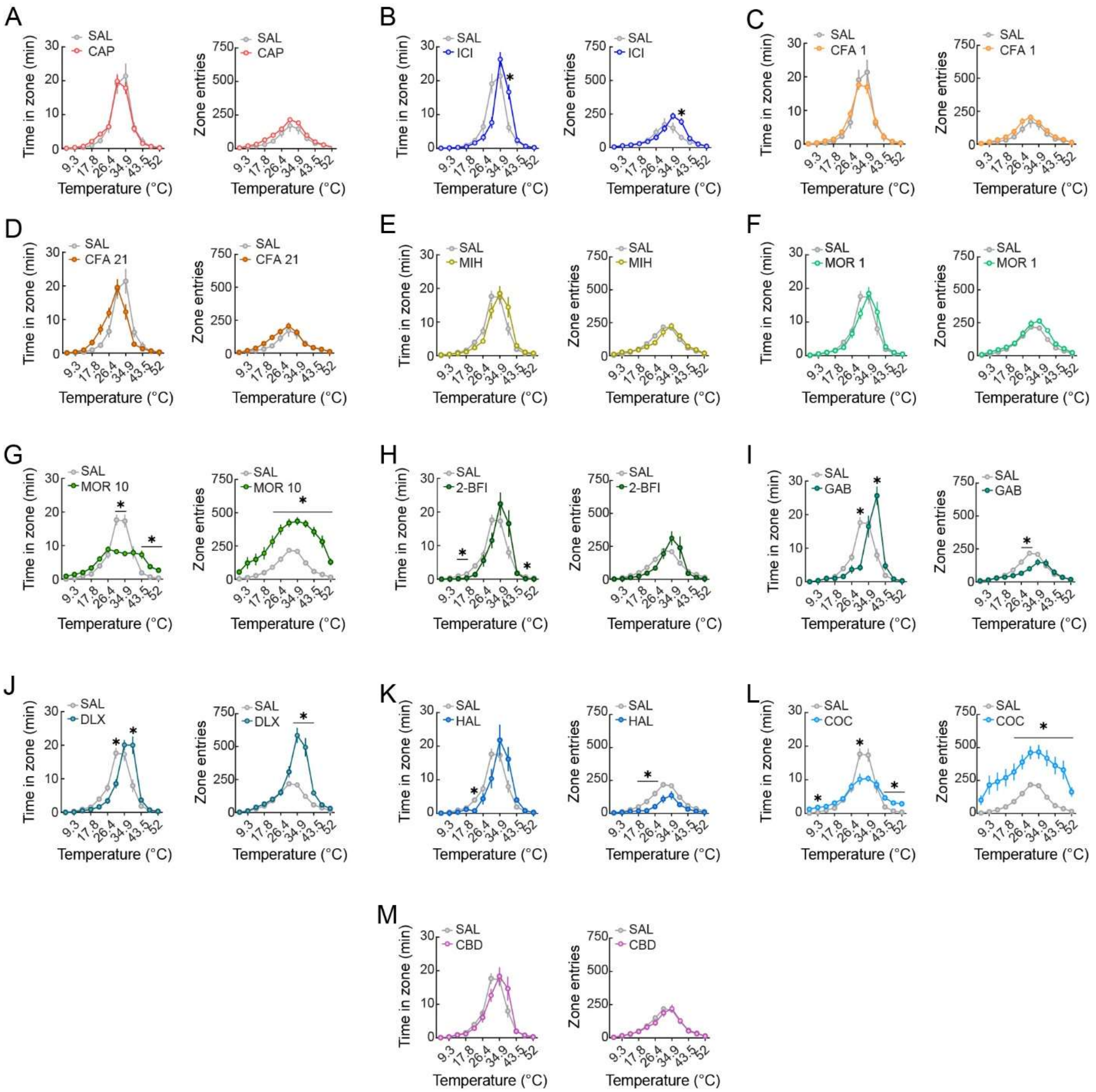

Fig. S3. Zone occupancy and zone entries using multiple comparisons tests.

(A–M) Summaries of zone occupancy and zone entries versus saline (SAL). Asterisks (*) indicate statistically significant effects in time spent in zone or zone entries by temperature zone, while black squares denote statistically significant timepoints. Two-way RM ANOVA with Šidák post-test ($P < 0.05$). Treatments: **(A)** CAP, capsaicin; **(B)** ICI, icilin; **(C)** CFA 1, Complete Freund's

Adjuvant 1 d post-injection; **(D)** CFA 21, Complete Freund's Adjuvant 21 d post-injection; **(E)** MIH, morphine-induced hyperalgesia; **(F)** MOR 1, 1 mg/kg morphine; **(G)** MOR 10, 10 mg/kg morphine; **(H)** 2-BFI, 2-BFI I_2R ligand; **(I)** GAB, gabapentin; **(J)** DLX, duloxetine; **(K)** HAL, haloperidol; **(L)** COC, cocaine; **(M)** CBD, cannabidiol. See **Table S1** for detailed statistics.

Figure S4

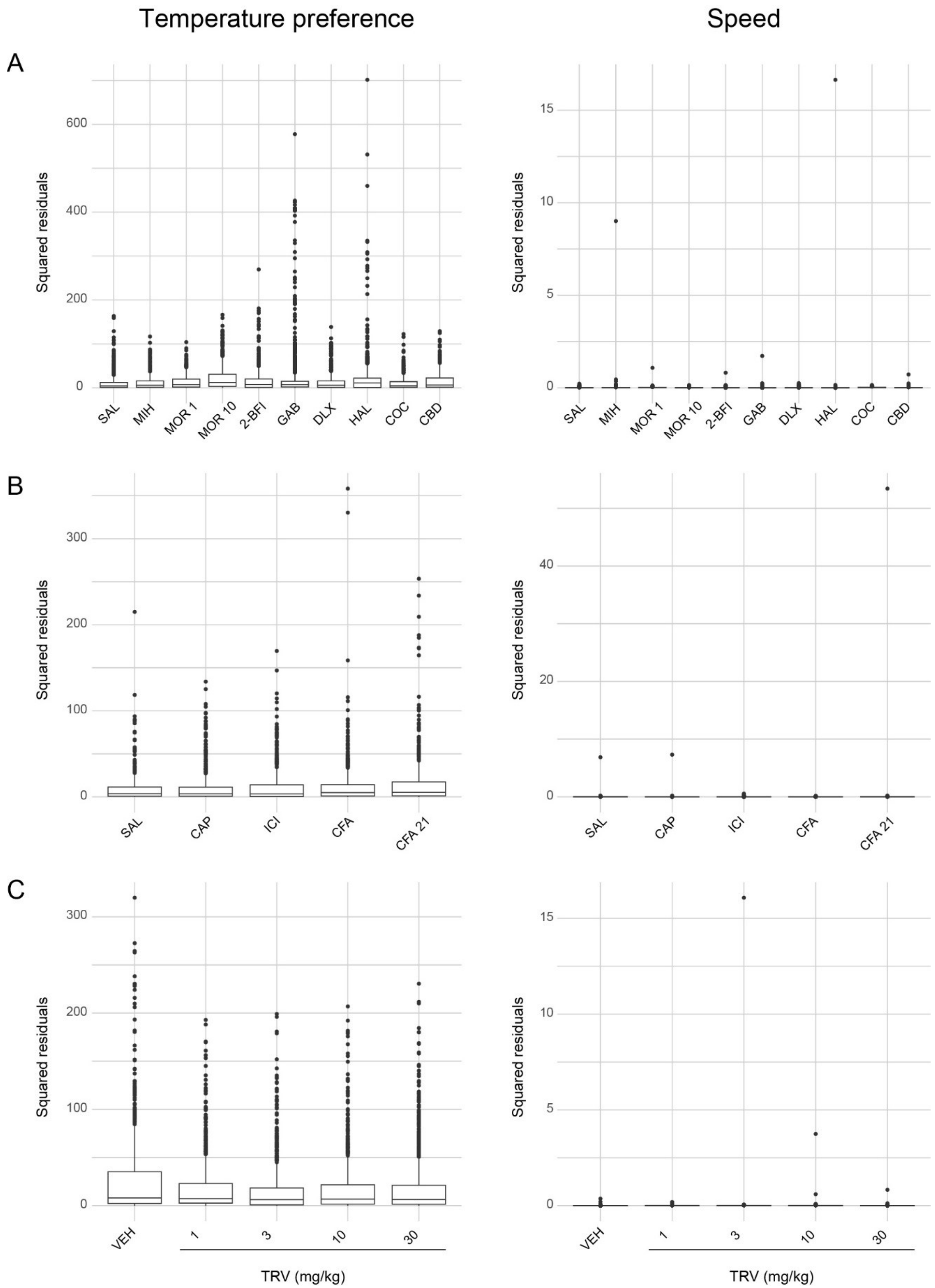
Temperature preference
Speed
A
B
C
Squared residuals
SAL
MIH
MOR 1
MOR 10
2-BFI
GAB
DLX
HAL
COC
CBD
CAP
ICI
CFA
CFA 21
VEH
1
3
10
30
TRV (mg/kg)

Fig. S4. Heteroscedasticity assessment using squared residuals from FLM models. (A–C) Squared residual plots from the FLM model testing treatments administered **(A)** intraperitoneally, **(B)** intraplantar, and **(C)** TRV734 doses. Residuals are pooled across animals and session timepoints. Each panel shows boxplots with individual points showing the distribution of squared residuals pooled across timepoints.

Figure S5

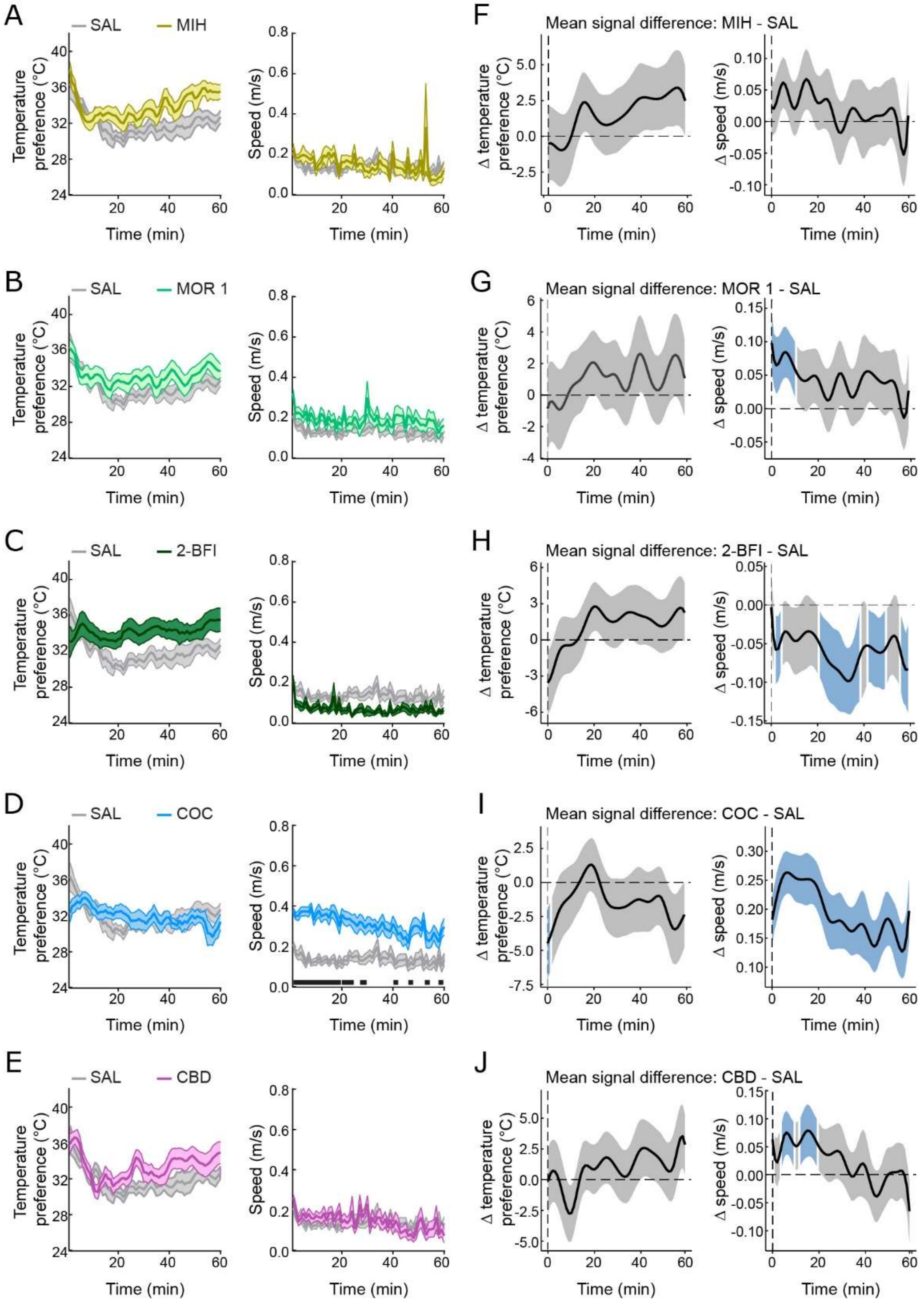
A
SAL
MIH
Temperature preference (°C)
Time (min)
Speed (m/s)
B
SAL
MOR 1
C
SAL
2-BFI
D
SAL
COC
E
SAL
CBD
F
Mean signal difference: MIH - SAL
Δ temperature preference (°C)
Δ speed (m/s)
G
Mean signal difference: MOR 1 - SAL
H
Mean signal difference: 2-BFI - SAL
I
Mean signal difference: COC - SAL
J
Mean signal difference: CBD - SAL

Fig. S5. FLM uncovers behavioral dynamics not captured by traditional time-course analyses. (A–F) Time-course plots of mean temperature preference (right) and speed for acute Complete Freund's Adjuvant (CFA 1), morphine-induced hyperalgesia (MIH), 1 mg/kg morphine (MOR 1), 2-BFI I_2R ligand (2-BFI), cocaine (COC), and cannabidiol (CBD). Black squares indicate statistically significant timepoints. Two-way RM-ANOVA (temperature preference) and mixed-effects RM models (speed), Šidák post hoc test ($P < 0.05$). Solid lines indicate the mean and shaded areas represent the s.e.m. calculated across animals at each timepoint. See **Table S1** for detailed statistics. **(G–L)** FLM coefficient estimates of the difference in average temperature (*left*) and speed (*right*) versus the control group (SAL). **(G)** CFA 1 promotes a significant preference for cooler zones and increases speed early in the session. **(H)** MIH does not evoke significant changes in temperature preference or speed. **(I)** MOR 1 shows a significant increase in speed during the initial minutes of the session. **(J)** 2-BFI decreases speed without affecting temperature preference. **(K)** COC administration significantly increases motor activity across the entire session but does not affect temperature preference. **(L)** CBD does not show alter temperature preference but slightly increases speed during the first 20 min.

Figure S6

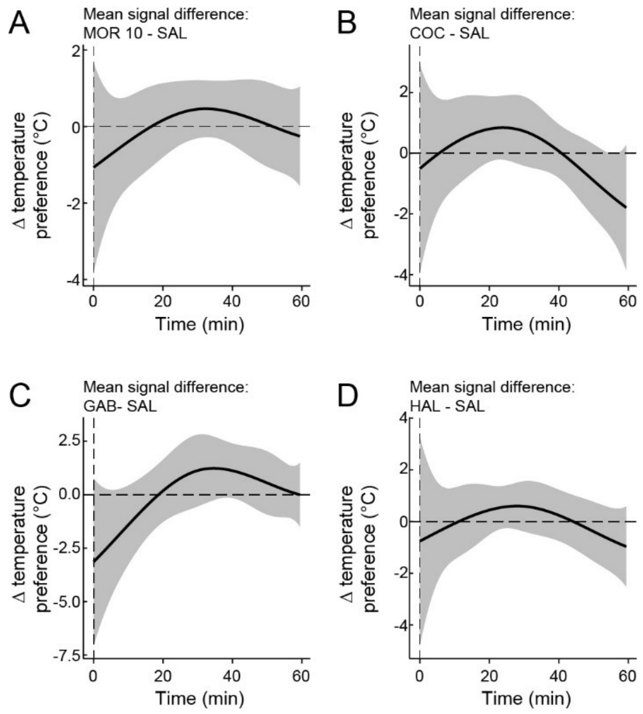

Fig. S6. FLM identifies compounds whose changes in temperature preference are potentially mediated by speed changes. (A−D) FLM coefficient estimates for temperature preference changes at a fixed speed versus saline (SAL) for **(A)** MOR 10, 10 mg/kg morphine, **(B)** COC, cocaine, **(C)** GAB, gabapentin, and **(D)** HAL, haloperidol. Smoothness was controlled with a roughness penalty (λ) with estimated values of λ = 11.86 for SAL, 540.57 for MOR 10, 9.16 for COC, 5.07 for GAB, and 39.02 for HAL.

Figure S7

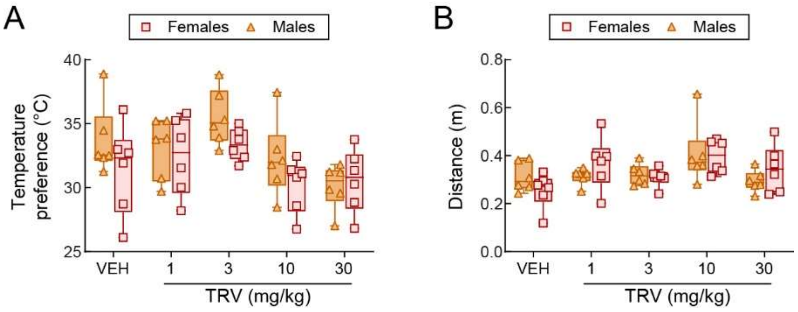

Fig. S7. TRV734 administration does not induce differences in temperature preference or locomotor activity between sexes.

(A) Temperature preference during a 2-h session after administration of vehicle (VEH) or TRV734 (TRV; 1 mg/kg to 30 mg/kg, i.p.) in female and male mice for each TRV dose. **(B)** Speed during a 2-h session after administration of VEH or TRV (1 mg/kg to 30 mg/kg, i.p.) in female and male mice across doses. Box plots show the median. See **Table S1** for detailed statistics.

Supplementary Methods

Experimental Treatments

Mice were tested with a range of treatments encompassing pain-promoting, analgesic, and non-pain-indicated conditions. TRPV1 and TRPM8 agonists included capsaicin (5 µg in a 10 µL volume, intraplantar [i.pl.]; *n* = 14) and icilin (30 µg in a 10 µL volume, i.pl.; *n* = 14). Capsaicin and icilin were dissolved in 80% DMSO in saline. Inflammatory pain was induced by a single injection of Complete Freund's Adjuvant (CFA; 20 µL of a 1:1 dilution in saline, i.pl.). CFA was tested on days 1 (acute, *n* = 14) and 21 (chronic, *n* = 14) post-injection (incomplete Freund's Adjuvant was not used as a control in this study). Control mice (SAL) were injected with 80% DMSO in saline (i.pl.). All intraplantar injections were performed under brief isoflurane anesthesia. Following intraplantar injections, mice were allowed to recover in an empty home cage for 5 min before testing. To reduce the total number of mice used, a single intraplantar-injected control group was used for all intraplantar-injected treatments.

Additional pronociceptive manipulation included morphine-induced hyperalgesia (3 days of twice-daily intraperitoneal [i.p.] injections of 10, 20, and 40 mg/kg morphine per injection ending the day before the test; *n* = 14). Analgesic compounds included morphine (1 and 10 mg/kg, i.p.; *n* = 14 and 20, respectively), gabapentin (100 mg/kg, i.p.; *n* = 14), 2-BFI (17.8 mg/kg, i.p.; *n* = 14), and TRV734 (1, 3, 10, and 30 mg/kg, i.p.; *n* = 12 per dose). Non-pain-indicated treatments included duloxetine (32 mg/kg, i.p.; *n* = 14), cocaine (15 mg/kg, i.p.; *n* = 15), haloperidol (0.56 mg/kg, i.p.; *n* = 14), and cannabidiol (56 mg/kg, i.p.; *n* = 14). For all intraperitoneally injected treatments, except TRV734, a single group of saline-injected mice (SAL, i.p.; *n* = 14) was used to reduce the total number of mice used in this study. For the novel

compound TRV734, a separate control group was injected with 25% (2-Hydroxypropyl)-β-cyclodextrin in water (i.p.; vehicle [VEH]; *n* = 12).

Duloxetine and haloperidol were dissolved in 10% DMSO in saline. TRV734 was dissolved in 25% (2-Hydroxypropyl)-β-cyclodextrin in water. Cannabidiol was dissolved in a 1:1:18 vehicle of ethanol:Tween-80:saline. The remaining drugs were dissolved in saline. All compounds were administered in a volume of 10 mL/kg. Cocaine, capsaicin, and icilin were injected 5 min prior to session start. TRV734 was injected immediately before the session. All other compounds were injected 30 min prior to the session. Mice underwent only 1 test.

Functional Linear Models

Functional Linear Models (FLM) were fit with the *fosr()* function [3; 4] and *pffr() function* [5] in the R package *refund* [2]. The first set of models are functional ANOVAs where the grouping factor is treatment group, and the functional outcome is either temperature or locomotion bin value. For example, for the analyses presented in **Fig. 2**, we fit the model with the following model code syntax:

$$model.fit = refund :: fosr(Y = as.matrix(preference.data),$$

$$X = model.matrix(pref \sim Drug, data = \ pref.dat))$$

where *preference.data* is a matrix, and the value in row *i* of column *s* contains the average temperature bin that animal *i* is in at session timepoint *s*. The *pref.data* dataframe contains the treatment group of each animal. This code fits the functional linear model indexed by session timepoints *s*:

$$Y_i(s) = \beta_0(s) \ + \sum_j Drug_{i,j}\, \beta_j(s) + \ \epsilon_i(s)$$

where $Y_i(s)$ is the average temperature value that animal *i* was in at session timepoint *s*. The variables $Drug_{i,j}$ are indicator variables of whether animal *i* received drug *j*. Saline is the reference group and so $\beta_0(s)$ represents the mean outcome of animals in the control group. The error of animal *i* at session timepoint *s* is denoted as $\epsilon_i(s)$. Each animal is in only one treatment group. We describe $Y_i(s)$ as the *average* temperature value that subject *i* was in at timepoint *s* because each timepoint *s* represents a 1-min time bin. Thus $Y_i(s)$ is the average value during this 1-min time bin.

The functional ANOVA model estimates the mean difference in temperature preferences of animals in a given treatment group relative to the baseline treatment group (saline) at each individual timepoint. The functional coefficient estimates, $\beta_1(s), \ldots, \beta_j(s)$, corresponding to each group indicator variable are the estimates for timepoint *s*.

The results presented in **Figs. 2, 3, and S5** are from two separate functional ANOVAs analyzing differences between specific compounds. We analyzed temperature preference as the functional outcome in the first analysis and locomotor speed as the functional outcome in the second functional ANOVA. Although the results are from two separate models, we present results side-by-side for each drug for ease of comparison. The temperature preference and locomotor speed vary over time, even in the baseline treatment group (saline). The corresponding curves for the other treatment groups display mean differences relative to this baseline.

For each model, we checked for heteroscedasticity in the outcome across treatment groups by examining the distribution of the squared residuals pooled across timepoints *s*. Most models showed signs of heteroscedasticity and so we fit a generalized least squares functional regression (GLS) with the *gls_cs()* function in the refund package [2]. Some GLS models failed to

fit due to singularity of the working covariance matrix. In these cases, we estimated coefficient estimate variance with a non-parametric bootstrap with 1000 replicates to ensure valid inference for the coefficient estimates. Finally, we used a Benjamini-Hochberg (BH) correction [1] to account for multiple comparisons of examining effects across the 60-min experiment. We show timepoints in which the effects are statistically significant after a BH correction by color coding the confidence interval bands in the functional coefficient plots.

The analyses presented in **Fig. S6** were *concurrent* FLM models akin to a concurrent functional ANCOVA. We fit these with the *pffr()* function in R. In addition to the treatment group factor variable, this model included the locomotion speed at each session timepoint as a functional covariate. This analysis was used to compare mean temperature preferences between groups, while adjusting for a linear and quadratic effect of locomotor activity (speed) at each time bin. The treatment factor variable compared saline group animals to morphine (10 mg/kg; MOR 10), cocaine, haloperidol, and 2-BFI. The functional coefficient $\beta_5(s)$ is the regression coefficient associated with the linear term for speed, $X_i(s)$, at timepoint *s*. The model is

$$Y_i(s) = \beta_0(s) + Mor_i\beta_1(s) + Coc_i\beta_2(s) + \ Hal_i\beta_3(s) + \ BFI_i\beta_4(s) +$$

$$X_i(s)\beta_5(s) \ + \ X_i^2(s)\beta_6(s) + \ \epsilon_i(s).$$

We used a non-parametric bootstrap to estimate functional coefficient estimate variance, followed by a BH correction.

References

[1] Benjamini Y, Hochberg Y. Controlling the False Discovery Rate - a Practical and Powerful Approach to Multiple Testing. J Roy Stat Soc B 1995;57(1):289–300.

[2] Goldsmith J SF, Huang L, Wrobel J, Di C, Gellar J, Harezlak J, McLean MW, Swihart B, Xiao L, Crainiceanu C, Reiss PT, Cui E refund: Regression with Functional Data, 2024.

[3] Ledoit O, Wolf M. Some hypothesis tests for the covariance matrix when the dimension is large compared to the sample size. Ann Stat 2002;30(4):1081–1102.

[4] Pourahmadi M. Joint mean-covariance models with applications to longitudinal data: Unconstrained parameterisation. Biometrika 1999;86(3):677–690.

[5] Scheipl F, Staicu AM, Greven S. Functional Additive Mixed Models. J Comput Graph Stat 2015;24(2):477–501.

Table S1. Statistical analysis details.

Figure	Test Name	Test Statistics	Test p Value	Multiple Comparisons	p Value
Figure 1E	Two-way RM ANOVA,	Temperature x Treatment: F (22, 495) = 6.261	$p < 0.0001$	Dunnett correction	
	SAL, MOR 1, MOR 10	Temperature: F (2.912, 131.0) = 87.64	$p < 0.0001$	for multiple comparisons	
		Treatment: F (2, 45) = 20.46	$p < 0.0001$	5: SAL vs. MOR 1	p = 0.1629
		Subject: F (45, 495) = 21.04	$p < 0.0001$	SAL vs. MOR 10	p = 0.0553
				9.3: SAL vs. MOR 1	p = 0.1401
				SAL vs. MOR 10	p = 0.0369
				13.5: SAL vs. MOR 1	p = 0.2297
				SAL vs. MOR 10	p = 0.0239
				17.8: SAL vs. MOR 1	p = 0.531
				SAL vs. MOR 10	p = 0.0103
				22.1: SAL vs. MOR 1	p = 0.9688
				SAL vs. MOR 10	p = 0.0011
				26.4: SAL vs. MOR 1	p = 0.8086
				SAL vs. MOR 10	p < 0.0001
				30.6: SAL vs. MOR 1	p = 0.6363
				SAL vs. MOR 10	p < 0.0001
				34.9: SAL vs. MOR 1	p = 0.0412
				SAL vs. MOR 10	p < 0.0001
				39.2: SAL vs. MOR 1	p = 0.0053
				SAL vs. MOR 10	p < 0.0001
				43.5: SAL vs. MOR 1	p = 0.1046
				SAL vs. MOR 10	p < 0.0001
				47.7: SAL vs. MOR 1	p = 0.4394
				SAL vs. MOR 10	p < 0.0001
				52: SAL vs. MOR 1	p = 0.5
				SAL vs. MOR 10	p = 0.0005
Figure 1F	Pearson's	SAL, $r^2 = 0.02426$	p = 0.5949		
	correlation	MOR 1, r2 = 0.05107	p = 0.4372		
	Zone Entries vs. Speed	MOR 10, r2 = 0.6621	p < 0.0001		
Figure 2A, left	One-way ANOVA	F (4, 57) = 14.90	p < 0.0001	Dunnett correction	
	Temperature Preference			for multiple comparisons	
				SAL vs. CAP	p = 0.4096
				SAL vs. ICI	p = 0.0424
				SAL vs. CFA	p = 0.6402
				SAL vs. CFA 21	p = 0.005

Figure 2A, right	One-way ANOVA<br>Speed	F (4, 57) = 0.3629	p = 0.8340		
Figure 2B, left	Two-way RM ANOVA,<br>Temperature Preference	Time x Treatment :F (59, 1062) = 2.034<br>Time: F (5.808, 104.5) = 1.680<br>Treatment: F (1, 18) = 3.286<br>Subject: F (18, 1062) = 12.33	p < 0.0001<br>p = 0.1354<br>p = 0.0866<br>p < 0.0001	Šídák correction<br>for multiple comparisons<br>SAL vs. CAP, significant minutes:	All ns
Figure 2B, right	Mixed-effects RM<br>Speed	Time x Treatment: F (59, 1061) = 1.671<br>Time: F (3.918, 70.46) = 2.034<br>Treatment: F (1, 18) = 0.05770	p = 0.0014<br>p =0.1002<br>*p = 0.8129*	Šídák correction<br>for multiple comparisons<br>SAL vs. CAP, significant minutes:	All ns
Figure 2C, left	Two-way RM ANOVA,<br>Temperature Preference	Time x Treatment :F (59, 1062) = 0.8053<br>Time: F (5.233, 94.19) = 1.481<br>Treatment: F (1, 18) = 6.854<br>Subject: F (18, 1062) = 22.59	p = 0.8534<br>p = 0.2009<br>p = 0.0174<br>p < 0.0001		
Figure 2C, right	Mixed-effects RM<br>Speed	Time x Treatment: F (59, 1062) = 2.117<br>Time: F (4.704, 84.67) = 1.940<br>Treatment: F (1, 18) = 0.002479	p =0.1002<br>p = 0.1003<br>p = 0.9608		
Figure 2D, left	Two-way RM ANOVA,<br>Temperature Preference	Time x Treatment :F (59, 1062) = 0.7699<br>Time: F (59, 1062) = 1.396<br>Treatment: F (1, 18) = 1.069<br>Subject: F (18, 1062) = 20.61	p = 0.8983<br>p = 0.0282<br>p = 0.3149<br>p < 0.0001		
Figure 2D, right	Mixed-effects RM<br>Speed	Time x Treatment: F (59, 1062) = 2.069<br>Time: F (3.848, 69.26) = 2.146<br>Treatment: F (1, 18) = 0.1412	p < 0.0001<br>p =0.0867<br>p = 0.7115	Šídák correction<br>for multiple comparisons<br>SAL vs. CFA 1, significant minutes:<br>31	p = 0.0438
Figure 2E, left	Two-way RM ANOVA,<br>Temperature Preference	Time x Treatment : F (59, 1062) = 1.270<br>Time: F (59, 1062) = 1.848<br>Treatment: F (1, 18) = 7.511<br>Subject: F (18, 1062) = 34.07	p = 0.0861<br>p = 0.0001<br>p = 0.0134<br>p < 0.0001	Šídák correction<br>for multiple comparisons<br>SAL vs. CFA 21, significant minutes:	All ns
Figure 2E, right	Mixed-effects RM<br>Speed	Time x Treatment: F (59, 1062) = 0.7530<br>Time: F (1.524, 27.44) = 0.7228	p =0.9162<br>p =0.4587		

		Treatment: F (1, 18) = 0.4115	p = 5293		
Figure 3A, left	One-way ANOVA<br>Temperature Preference	F (9, 137) = 3.344	p = 0.0010	Dunnett correction<br>for multiple comparisons	
				SAL vs. MIH	p = 0.3716
				SAL vs. MOR 1	p = 0.8277
				SAL vs. MOR 10	p > 0.9999
				SAL vs. 2-BFI	p = 0.203
				SAL vs. GAB	p = 0.0059
				SAL vs. DLX	p = 0.0222
				SAL vs. HAL	p = 0.0536
				SAL vs. COC	p > 0.9999
				SAL vs. CBD	p = 0.7204
Figure 3A, right	One-way ANOVA<br>Speed	F (9, 137) = 23.04	p < 0.0001	Dunnett correction<br>for multiple comparisons	
				SAL vs. MIH	p = 0.9965
				SAL vs. MOR 1	p = 0.1487
				SAL vs. MOR 10	p = 0.0078
				SAL vs. 2-BFI	p = 0.0408
				SAL vs. GAB	p = 0.0375
				SAL vs. DLX	p > 0.9999
				SAL vs. HAL	p = 0.0033
				SAL vs. COC	p < 0.0001
				SAL vs. CBD	p > 0.9999
Figure 3B, left	Two-way RM ANOVA,<br>Temperature Preference	Time x Treatment : F (59, 1888) = 1.350<br>Time: F (5.912, 189.2) = 3.991<br>Treatment: F (1, 32) = 0.05914<br>Subject: F (32, 1888) = 109.8	p = 0.0409<br>p = 0.0009<br>p = 0.8094<br>p < 0.0001	Šídák correction<br>for multiple comparisons<br>SAL vs. MOR 10, significant minutes:	All ns
Figure 3B, right	Mixed-effects RM<br>Speed	Time x Treatment: F (59, 1888) = 1.200<br>Time: F (11.50, 367.8) = 2.434<br>Treatment: F (1, 32) = 9.938	p = 0.1448<br>p =0.0054<br>p = 0.0035	Šídák correction<br>for multiple comparisons<br>SAL vs. MOR 10, significant minutes:	All ns
Figure 3C, left	Two-way RM ANOVA,<br>Temperature Preference	Time x Treatment : F (59, 1534) = 2.231<br>Time: F (6.317, 164.2) = 1.795<br>Treatment: F (1, 26) = 12.32<br>Subject: F (26, 1534) = 44.04	p < 0.0001<br>p = 0.0991<br>p = 0.0017<br>p < 0.0001	Šídák correction<br>for multiple comparisons<br>SAL vs. GAB, significant minutes:	
				18	p = 0.0194
				19	p = 0.0073
				20	p = 0.0016
				21	p = 0.0009

				22	p = 0.0001
				23	p = 0.0006
				24	p = 0.0165
				25	p = 0.0415
				26	p = 0.048
				27	p = 0.0158
				28	p = 0.0232
				29	p = 0.0017
				30	p = 0.0012
				31	p = 0.004
				32	p = 0.0132
				33	p = 0.0028
				34	p = 0.0165
				46	p = 0.0125
				47	p = 0.0133
				48	p = 0.0462
Figure 3C, right	Mixed-effects RM	Time x Treatment: F (59, 1519) = 1.447	p = 0.0160	Šídák correction	
	Speed	Time: F (10.41, 268.0) = 2.561	p =0.0050	for multiple comparisons	
		Treatment: F (1, 26) = 15.35	p = 0.0006	SAL vs. GAB, significant minutes:	
				33	p = 0.0360
				34	p = 0.0281
				41	p =0.0298
				45	p =0.0327
Figure 3D, left	Two-way RM ANOVA,	Time x Treatment : F (59, 1534) = 0.7657	p = 0.9043	Šídák correction	
	Temperature Preference	Time: F (8.684, 225.8) = 4.732	p < 0.0001	for multiple comparisons	
		Treatment: F (1, 26) = 17.20	p = 0.0003	SAL vs. DLX, significant minutes:	All ns
		Subject: F (26, 1534) = 34.19	p < 0.0001		
Figure 3D, right	Mixed-effects RM	Time x Treatment: F (59, 1534) = 0.9523	p = 0.5794	Šídák's correction	
	Speed	Time: F (14.19, 368.8) = 1.794	p = 0.037	for multiple comparisons	
		Treatment: F (1, 26) = 0.009322	p = 0.9238	SAL vs. DLX, significant minutes:	All ns
Figure 3E, left	Two-way RM ANOVA,	Time x Treatment : F (59, 1534) = 0.5420	p = 0.9982	Šídák correction	
	Temperature Preference	Time: F (6.677, 173.6) = 3.165	p = 0.0041	for multiple comparisons	
		Treatment: F (1, 26) = 8.161	p = 0.0083	SAL vs. HAL, significant minutes:	All ns
		Subject: F (26, 1534) = 43.31	p < 0.0001		
Figure 3E, right	Mixed-effects RM	Time x Treatment: F (59, 1518) = 1.034	p = 0.4062	Šídák correction	
	Speed	Time: F (2.122, 54.61) = 1.605	p = 0.2090	for multiple comparisons	

		Treatment: F (1, 26) = 30.40	p < 0.0001	SAL vs. HAL, significant minutes:	All ns
Figure 4A	One-way ANOVA<br>Total Zone Entries	F (4, 55) = 34.66	p < 0.0001	Dunnett correction<br>for multiple comparisons	
				VEH vs. TRV 1	p = 0.2536
				VEH vs. TRV 3	p = 0.0012
				VEH vs. TRV 10	p < 0.0001
				VEH vs. TRV 30	p < 0.0001
Figure 4B	Two-way RM ANOVA,<br>TRV734- Zone Entries	Temperature x Treatment: F (44, 605) = 5.374	*p* < 0.0001	Dunnett correction	
		Temperature: F (2.501, 137.6) = 95.13	*p* < 0.0001	for multiple comparisons	
		Treatment: F (4, 55) = 34.66	*p* < 0.0001	5: VEH vs. TRV 1	p = 0.7312
		Subject: F (55, 605) = 7.794	*p* < 0.0001	VEH vs. TRV 3	p = 0.9949
				VEH vs. TRV 10	p = 0.0495
				VEH vs. TRV 30	p < 0.0001
				9.3: VEH vs. TRV 1	p = 0.6519
				VEH vs. TRV 3	p = 0.9604
				VEH vs. TRV 10	p = 0.0152
				VEH vs. TRV 30	p < 0.0001
				13.5: VEH vs. TRV 1	p = 0.6092
				VEH vs. TRV 3	p = 0.8355
				VEH vs. TRV 10	p = 0.0008
				VEH vs. TRV 30	p < 0.0001
				17.8: VEH vs. TRV 1	p = 0.4718
				VEH vs. TRV 3	p = 0.4013
				VEH vs. TRV 10	p < 0.0001
				VEH vs. TRV 30	p < 0.0001
				22.1: VEH vs. TRV 1	p = 0.3329
				VEH vs. TRV 3	p = 0.1302
				VEH vs. TRV 10	<0.0001
				VEH vs. TRV 30	<0.0001
				26.4: VEH vs. TRV 1	p = 0.2398
				VEH vs. TRV 3	p = 0.0491
				VEH vs. TRV 10	p = 0.0001
				VEH vs. TRV 30	p < 0.0001
				30.6: VEH vs. TRV 1	p = 0.2355
				VEH vs. TRV 3	p = 0.0211
				VEH vs. TRV 10	p = 0.0004
				VEH vs. TRV 30	p = 0.0016
				34.9: VEH vs. TRV 1	p = 0.02

				VEH vs. TRV 3	p = 0.0005
				VEH vs. TRV 10	p = 0.0002
				VEH vs. TRV 30	p < 0.0001
				39.2: VEH vs. TRV 1	p = 0.0179
				VEH vs. TRV 3	p = 0.0009
				VEH vs. TRV 10	p < 0.0001
				VEH vs. TRV 30	p < 0.0001
				43.5: VEH vs. TRV 1	p = 0.0279
				VEH vs. TRV 3	p = 0.0178
				VEH vs. TRV 10	p < 0.0001
				VEH vs. TRV 30	p < 0.0001
				47.7: VEH vs. TRV 1	p = 0.0942
				VEH vs. TRV 3	p = 0.1441
				VEH vs. TRV 10	p = 0.0002
				VEH vs. TRV 30	p < 0.0001
				52: VEH vs. TRV 1	p = 0.2579
				VEH vs. TRV 3	p = 0.2418
				VEH vs. TRV 10	p = 0.0009
				VEH vs. TRV 30	p < 0.0001
Figure 4C	Two-way RM ANOVA,	Temperature x Treatment: F (44, 605) = 4.930	*p* < 0.0001	Dunnett correction	
	TRV734-Time in Zone	Temperature: F (2.509, 138.0) = 78.78	*p* < 0.0001	for multiple comparisons	
		Treatment: F (4, 55) = 1.016	p = 0.0474	5: VEH vs. TRV 1	p = 0.9516
		Subject: F (55, 605) = 1.144e-005	p > 0.9999	VEH vs. TRV 3	p = 0.9957
				VEH vs. TRV 10	p = 0.0337
				VEH vs. TRV 30	p < 0.0001
				9.3: VEH vs. TRV 1	p = 0.9061
				VEH vs. TRV 3	p > 0.9999
				VEH vs. TRV 10	p = 0.0052
				VEH vs. TRV 30	p < 0.0001
				13.5: VEH vs. TRV 1	p = 0.8944
				VEH vs. TRV 3	p > 0.9999
				VEH vs. TRV 10	p = 0.0002
				VEH vs. TRV 30	p < 0.0001
				17.8: VEH vs. TRV 1	p > 0.9999
				VEH vs. TRV 3	p = 0.9543
				VEH vs. TRV 10	p = 0.0209
				VEH vs. TRV 30	p < 0.0001
				22.1: VEH vs. TRV 1	p = 0.9994

				VEH vs. TRV 3	p = 0.9817
				VEH vs. TRV 10	p = 0.8106
				VEH vs. TRV 30	p = 0.4604
				26.4: VEH vs. TRV 1	p = 0.9975
				VEH vs. TRV 3	p = 0.485
				VEH vs. TRV 10	p = 0.9993
				VEH vs. TRV 30	p = 0.497
				30.6: VEH vs. TRV 1	p = 0.9937
				VEH vs. TRV 3	p = 0.408
				VEH vs. TRV 10	p = 0.3435
				VEH vs. TRV 30	p = 0.0137
				34.9: VEH vs. TRV 1	p > 0.9999
				VEH vs. TRV 3	p = 0.9548
				VEH vs. TRV 10	p = 0.0173
				VEH vs. TRV 30	p = 0.0015
				39.2: VEH vs. TRV 1	p = 0.9998
				VEH vs. TRV 3	p = 0.6373
				VEH vs. TRV 10	p = 0.9911
				VEH vs. TRV 30	p = 0.8318
				43.5: VEH vs. TRV 1	p = 0.888
				VEH vs. TRV 3	p = 0.0577
				VEH vs. TRV 10	p = 0.004
				VEH vs. TRV 30	p < 0.0001
				47.7: VEH vs. TRV 1	p = 0.1135
				VEH vs. TRV 3	p = 0.0835
				VEH vs. TRV 10	p = 0.0002
				VEH vs. TRV 30	p < 0.0001
				52: VEH vs. TRV 1	p = 0.6993
				VEH vs. TRV 3	p = 0.2701
				VEH vs. TRV 10	p = 0.0015
				VEH vs. TRV 30	p < 0.0001
Figure 4D, left	One-way ANOVA	F (4, 55) = 4.501	p = 0.0032	Dunnett correction	
	Temperature Preference			for multiple comparisons	
				VEH vs. TRV1	p = 0.9986
				VEH vs. TRV3	p = 0.2285
				VEH vs. TRV10	p = 0.523
				VEH vs. TRV30	p = 0.1212
Figure 4D, right	One-way ANOVA	F (4, 55) = 3.990	p = 0.0065	Dunnett correction	

	Speed			for multiple comparisons	
				VEH vs. TRV1	p = 0.1896
				VEH vs. TRV3	p = 0.7
				VEH vs. TRV10	p = 0.0014
				VEH vs. TRV30	p = 0.5897
Figure 4E, left	Two-way RM ANOVA,	Time x Treatment : F (119, 2618) = 1.494	p = 0.0006	Šídák correction	
	Temperature Preference	Time: F (11.03, 242.7) = 0.6615	p = 0.7746	for multiple comparisons	
		Treatment: F (1, 22) = 0.03540	p = 0.8525	VEH vs. TRV 1, significant minutes:	All ns
		Subject: F (22, 2618) = 81.10	p < 0.0001		
Figure 4E right	Mixed-effects RM	Time x Treatment: F (119, 2612) = 1.091	p = 0.5794		
	Speed	Time: F (8.880, 194.9) = 4.035	p = 0.2409		
		Treatment: F (1, 22) = 9.685	p = 0.0051		
Figure 4F, left	Two-way RM ANOVA,	Time x Treatment : F (119, 2618) = 1.094	p = 0.2337		
	Temperature Preference	Time: F (7.766, 170.9) = 0.9374	p = 0.4854		
		Treatment: F (1, 22) = 2.916	p = 0.1018		
		Subject: F (22, 2618) = 70.44	p < 0.0001		
Figure 4F right	Mixed-effects RM	Time x Treatment: F (119, 2614) = 1.688	p < 0.0001	Šídák correction	
	Speed	Time: F (2.207, 48.48) = 3.211	p = 0.0444	for multiple comparisons	
		Treatment: F (1, 22) = 9.685	p = 0.0001	VEH vs. TRV 3, significant minutes:	
				9	p = 0.0185
				10	p = 0.0148
				11	p = 0.0126
				13	p = 0.0033
				14	p = 0.0061
				15	p = 0.004
				16	p = 0.0006
				17	p = 0.0002
				18	p = 0.0095
				19	p = 0.0021
				21	p = 0.0088
				22	p = 0.0263
				23	p = 0.0006
				24	p = 0.0091
				25	p = 0.0097
				26	p = 0.0383
				27	p = 0.0005

				28	p = 0.0315
				29	p = 0.0103
				32	p = 0.0413
				55	p = 0.0028
Figure 4G, left	Two-way RM ANOVA,	Time x Treatment : F (119, 2618) = 1.181	p = 0.0922		
	Temperature Preference	Time: F (9.831, 216.3) = 3.297	p = 0.0006		
		Treatment: F (1, 22) = 1.210	p = 0.2832		
		Subject: F (22, 2618) = 86.21	p < 0.0001		
Figure 4G right	Mixed-effects RM	Time x Treatment: F (119, 2614) = 1.710	p < 0.0001	Šídák correction	
	Speed	Time: F (6.885, 151.2) = 4.409	p = 0.0002	for multiple comparisons	
		Treatment: F (1, 22) = 52.96	p < 0.0001	VEH vs. TRV 10, significant minutes:	
				4	p = 0.0011
				5	p = 0.0015
				6	p = 0.0005
				7	p = 0.0036
				8	p = 0.016
				9	p = 0.0006
				10	p < 0.0001
				11	p = 0.0039
				12	p = 0.003
				13	p = 0.0003
				14	p < 0.0001
				15	p = 0.0001
				16	p = 0.0059
				17	p = 0.0018
				18	p = 0.0283
				19	p = 0.0041
				21	p = 0.0006
				22	p = 0.0052
				23	p = 0.0046
				24	p = 0.0038
				25	p = 0.0016
				26	p = 0.0025
				27	p = 0.0008
				28	p = 0.0102
				29	p = 0.0083
				30	p = 0.0493

				31	p = 0.0001
				32	p = 0.0035
				33	p = 0.0037
				34	p = 0.0082
				35	p = 0.0017
				36	p = 0.0005
				37	p = 0.0038
				38	p = 0.0108
				42	p = 0.0074
				47	p = 0.0189
				55	p = 0.0104
				56	p = 0.03
				57	p = 0.0127
				59	p = 0.0278
				61	p = 0.0255
				62	p = 0.0002
				63	p = 0.0009
				64	p = 0.0049
				65	p = 0.0106
				66	p = 0.0009
				67	p < 0.0001
				68	p = 0.0001
				69	p = 0.0004
				70	p = 0.038
				72	p = 0.027
				75	p = 0.0002
				76	p = 0.0117
				84	p = 0.0284
Figure 4H, left	Two-way RM ANOVA,	Time x Treatment : F (119, 2618) = 1.008	p = 0.4587		
	Temperature Preference	Time: F (8.772, 193.0) = 2.537	p = 0.0096		
		Treatment: F (1, 22) = 4.006	p = 0.0578		
		Subject: F (22, 2618) = 60.00	p < 0.0001		
Figure 4H right	Mixed-effects RM	Time x Treatment: F (119, 2614) = 1.768	p < 0.0001	Šídák correction	
	Speed	Time: F (119, 2614) = 5.699	p < 0.0001	for multiple comparisons	
		Treatment: F (1, 22) = 51.70	p < 0.0001	VEH vs. TRV 30, significant minutes:	
				3	p < 0.0001
				4	p < 0.0001

				5	p < 0.0001
				6	p = 0.0011
				10	p = 0.0356
				11	p < 0.0001
				13	p = 0.0028
				63	p = 0.0482
				64	p = 0.0265
				65	p = 0.0027
				66	p = 0.0248
				67	p = 0.0038
				68	p = 0.0482
				69	p = 0.0126
				70	p = 0.0106
				72	p = 0.0035
				73	p = 0.0012
				74	p = 0.0039
				75	p = 0.0003
				76	p < 0.0001
				77	p = 0.0011
				79	p = 0.0029
				80	p = 0.0434
				81	p = 0.006
				82	p = 0.0052
				84	p < 0.0001
				85	p = 0.0048
				86	p = 0.0024
				87	p = 0.014
				89	p = 0.0007
				90	p < 0.0001
				91	p = 0.0005
				92	p = 0.0059
				93	p = 0.0412
				94	p = 0.0037
				96	p = 0.0312
				103	p = 0.0258
				104	p = 0.0338
				105	p = 0.0004
				108	p = 0.0083

				109	p = 0.0376
				112	p = 0.028
				113	p = 0.0028
				115	p = 0.0059
				116	p = 0.0034
				118	p = 0.002
				119	p = 0.0023
				120	p = 0.0412
Figure S1A	Unpaired t-test<br>Temperature Preference	t(18) = 0.7267	p = 0.4768		
Figure S1B	Unpaired t-test<br>Speed	t(18) = 0.4548	p = 0.6547		
Figure S2A	One-way ANOVA<br>Temperature Preference	F (2, 31) = 10.65	p = 0.0003	Dunnett correction<br>for multiple comparisons<br>SAL vs. CAP<br>SAL vs. ICI	 <br>p = 0.0209<br>p = 0.5901
Figure S2B	One-way ANOVA<br>Temperature Preference	F (2, 31) = 7.026	p = 0.0030	Dunnett correction<br>for multiple comparisons<br>SAL vs. CAP<br>SAL vs. ICI	 <br>p = 0.2479<br>p = 0.2382
Figure S2C	One-way ANOVA<br>Temperature Preference	F (2, 31) = 10.05	p = 0.0004	Dunnett correction<br>for multiple comparisons<br>SAL vs. CAP<br>SAL vs. ICI	 <br>p = 0.0572<br>p = 0.3433
Figure S2D	One-way ANOVA<br>Temperature Preference	F (2, 31) = 0.5816	p = 0.5650		
Figure S2E	One-way ANOVA<br>Temperature Preference	F (2, 31) = 5.464	p = 0.0093	Dunnett correction<br>for multiple comparisons<br>SAL vs. CAP<br>SAL vs. ICI	 <br>p = 0.4549<br>p = 0.0102
Figure S2F	One-way ANOVA<br>Temperature Preference	F (2, 31) = 11.73	p = 0.0002	Dunnett correction<br>for multiple comparisons<br>SAL vs. CAP<br>SAL vs. ICI	 <br>p = 0.3995<br>p = 0.0259
Figure S2G	One-way ANOVA<br>Temperature Preference	F (2, 31) = 11.91	p = 0.0001	Dunnett correction<br>for multiple comparisons	

				SAL vs. CAP<br>SAL vs. ICI	p = 0.2359<br>p = 0.0485
Figure S2H	One-way ANOVA<br>Temperature Preference	F (2, 31) = 7.718	p = 0.0019	Dunnett correction<br>for multiple comparisons<br>SAL vs. CAP<br>SAL vs. ICI	 <br>p = 0.1322<br>p = 0.3408
Figure S3A, left<br><br><br><br><br>Figure S3A, right	Two-way RM ANOVA,<br>Time in Zone<br><br><br><br>Two-way RM ANOVA,<br>Zone entries	Time x Treatment : F (11, 198) = 0.5750<br>Time: F (2.095, 37.71) = 69.70<br>Treatment: F (1, 18) = 0.1248<br>Subject: F (18, 198) = 1.762e-008<br><br>Time x Treatment: F (11, 198) = 1.041<br>Time: F (2.677, 48.18) = 50.45<br>Treatment: F (1, 18) = 1.735<br>Subject: F (18, 198) = 9.624	p = 0.8478<br>p < 0.0001<br>p = 0.728<br>p > 0.9999<br><br>p = 0.4116<br>p < 0.0001<br>p = 0.2042<br>p < 0.0001		
Figure S3B, left<br><br><br><br><br>Figure S3B, right	Two-way RM ANOVA,<br>Time in Zone<br><br><br><br>Two-way RM ANOVA,<br>Zone entries	Time x Treatment : F (11, 198) = 6.209<br>Time: F (2.317, 41.70) = 54.80<br>Treatment: F (1, 18) = 0.1636<br>Subject: F (18, 198) = 9.673e-009<br><br>Time x Treatment: F (11, 198) = 4.624<br>Time: F (2.449, 44.08) = 35.55<br>Treatment: F (1, 18) = 0.5295<br>Subject: F (18, 198) = 8.138	p < 0.0001<br>p < 0.0001<br>p = 0.6906<br>p > 0.9999<br><br>p < 0.0001<br>p < 0.0001<br>p = 0.4762<br>p < 0.0001	Šídák correction<br>for multiple comparisons<br>SAL vs. ICI, significant temperature:<br>39.2<br><br>Šídák correction<br>for multiple comparisons<br>SAL vs. ICI, significant temperature:<br>39.2	 <br><br>p = 0.0113<br><br><br><br><br>p = 0.0259
Figure S3C, left<br><br><br><br><br>Figure S3C, right	Two-way RM ANOVA,<br>Time in Zone<br><br><br><br>Two-way RM ANOVA,<br>Zone entries	Time x Treatment : F (11, 198) = 0.8891<br>Time: F (2.129, 38.33) = 60.89<br>Treatment: F (1, 18) = 1.652<br>Subject: F (18, 198) = 1.995e-008<br><br>Time x Treatment: F (11, 198) = 0.9469<br>Time: F (2.129, 38.32) = 45.02<br>Treatment: F (1, 18) = 1.551<br>Subject: F (18, 198) = 9.038	p = 0.552<br>p < 0.0001<br>p = 0.2149<br>p > 0.9999<br><br>p = 0.4966<br>p < 0.0001<br>p = 0.2289<br>p < 0.0001		
Figure S3D, left	Two-way RM ANOVA,<br>Time in Zone	Time x Treatment : F (11, 198) = 2.906<br>Time: F (2.199, 39.58) = 37.09<br>Treatment: F (1, 18) = 1.194<br>Subject: F (18, 198) = 5.850e-009	p = 0.0014<br>p < 0.0001<br>p = 0.2890<br>p > 0.9999	Šídák correction<br>for multiple comparisons<br>SAL vs. CFA 21, significant temperature:	 <br>All ns

Figure S3D, right	Two-way RM ANOVA,<br>Zone entries	Time x Treatment: F (11, 198) = 1.193<br>Time: F (2.215, 39.88) = 32.85<br>Treatment: F (1, 18) = 1.151<br>Subject: F (18, 198) = 7.770	p = 0.2939<br>p < 0.0001<br>p = 0.2975<br>p < 0.0001		
Figure S3E, left	Two-way RM ANOVA,<br>Time in Zone	Time x Treatment : F (11, 286) = 2.230<br>Time: F (2.384, 62.00) = 52.66<br>Treatment: F (1, 26) = 0.02820<br>Subject: F (26, 286) = 1.382e-008	p = 0.0132<br>p < 0.0001<br>p = 0.8679<br>p > 0.9999	Šídák correction<br>for multiple comparisons<br>SAL vs. MIH, significant temperature:	 <br>All ns
Figure S3E, right	Two-way RM ANOVA,<br>Zone entries	Time x Treatment: F (11, 286) = 2.100<br>Time: F (2.434, 63.29) = 73.24<br>Treatment: F (1, 26) = 0.02391<br>Subject: F (26, 286) = 5.757	p = 0.0204<br>p < 0.0001<br>p = 0.8783<br>p < 0.0001	Šídák correction<br>for multiple comparisons<br>SAL vs. MIH, significant temperature:	 <br>All ns
Figure S3F, left	Two-way RM ANOVA,<br>Time in Zone	Time x Treatment : F (11, 286) = 1.828<br>Time: F (2.040, 53.04) = 55.58<br>Treatment: F (1, 26) = 0.7573<br>Subject: F (26, 286) = 2.022e-008	p = 0.0491<br>p < 0.0001<br>p = 0.3921<br>p > 0.9999	Šídák correction<br>for multiple comparisons<br>SAL vs. MOR 1, significant temperature:	 <br>All ns
Figure S3F, right	Two-way RM ANOVA,<br>Zone entries	Time x Treatment: F (11, 286) = 1.550<br>Time: F (2.150, 55.90) = 108.3<br>Treatment: F (1, 26) = 3.663<br>Subject: F (26, 286) = 7.116	p = 0.1133<br>p < 0.0001<br>p = 0.0667<br>p < 0.0001		
Figure S3G, left	Two-way RM ANOVA,<br>Time in Zone	Time x Treatment : F (11, 352) = 13.38<br>Time: F (3.600, 115.2) = 47.66<br>Treatment: F (1, 32) = 0.007488<br>Subject: F (32, 352) = 1.550e-008	p < 0.0001<br>p < 0.0001<br>p = 0.9316<br>p > 0.9999	Šídák correction<br>for multiple comparisons<br>SAL vs. MOR 10, significant temperature:<br>30.6<br>34.9<br>43.5<br>47.7<br>52	 <br><br>p = 0.0006<br>p = 0.0031<br>p = 0.0033<br>p < 0.0001<br>p = 0.0015
Figure S3F, right	Two-way RM ANOVA,<br>Zone entries	Time x Treatment: F (11, 352) = 8.173<br>Time: F (2.591, 82.92) = 56.40<br>Treatment: F (1, 32) = 24.20<br>Subject: F (32, 352) = 23.13	p < 0.0001<br>p < 0.0001<br>p < 0.0001<br>p < 0.0001	Šídák correction<br>for multiple comparisons<br>SAL vs. MOR 10, significant temperature:<br>22.1<br>26.4<br>30.6<br>34.9<br>39.2	 <br><br>p = 0.0069<br>p = 0.0003<br>p < 0.0001<br>p < 0.0001<br>p < 0.0001

				43.5	p < 0.0001
				47.7	p = 0.0003
				52	p = 0.0033
Figure S3H, left	Two-way RM ANOVA,	Time x Treatment : F (11, 286) = 3.022	p = 0.0008	Šídák correction	
	Time in Zone	Time: F (2.293, 59.61) = 41.45	p < 0.0001	for multiple comparisons	
		Treatment: F (1, 26) = 0.03485	p = 0.8534	SAL vs. 2-BFI, significant temperature:	
		Subject: F (26, 286) = 7.546e-009	p > 0.9999	13.5	p = 0.0063
				17.8	p = 0.0005
				47.7	p = 0.0166
Figure S3H, right	Two-way RM ANOVA,	Time x Treatment: F (11, 286) = 2.827	p =0.0016	Šídák correction	
	Zone entries	Time: F (2.228, 57.94) = 29.75	p < 0.0001	for multiple comparisons	
		Treatment: F (1, 26) = 0.008165	p = 0.9287	SAL vs. 2-BFI, significant temperature:	All ns
		Subject: F (26, 286) = 2.174	p = 0.0011		
Figure S3I, left	Two-way RM ANOVA,	Time x Treatment : F (11, 286) = 14.64	p < 0.0001	Šídák correction	
	Time in Zone	Time: F (2.489, 64.72) = 49.61	p < 0.0001	for multiple comparisons	
		Treatment: F (1, 26) = 1.040	p = 0.3172	SAL vs. GAB, significant temperature:	
		Subject: F (26, 286) = 1.268e-008	p > 0.9999	30.6	p < 0.0001
				39.2	p = 0.0003
Figure S3I, right	Two-way RM ANOVA,	Time x Treatment: F (11, 286) = 8.204	p < 0.0001	Šídák correction	
	Zone entries	Time: F (26, 286) = 8.116	p < 0.0001	for multiple comparisons	
		Treatment: F (1, 26) = 0.008165	p = 0.0847	SAL vs. GAB, significant temperature:	
		Subject: F (26, 286) = 2.174	p < 0.0001	26.4	p = 0.0197
				30.6	p = 0.0012
Figure S3J, left	Two-way RM ANOVA,	Time x Treatment : F (11, 286) = 10.66	p < 0.0001	Šídák correction	
	Time in Zone	Time: F (2.168, 56.37) = 76.11	p < 0.0001	for multiple comparisons	
		Treatment: F (1, 26) = 0.6757	p = 0.4186	SAL vs. DLX, significant temperature:	
		Subject: F (26, 286) = 1.972e-008	p > 0.9999	30.6	p = 0.0029
				39.2	p = 0.0117
Figure S3J, right	Two-way RM ANOVA,	Time x Treatment : F (11, 286) = 19.40	p < 0.0001	Šídák correction	
	Zone entries	Time: F (2.273, 59.10) = 67.79	p < 0.0001	for multiple comparisons	
		Treatment: F (26, 286) = 2.147	p < 0.0001	SAL vs. DLX, significant temperature:	
		Subject: F (26, 286) = 1.268e-008	p = 0.0013	34.9	p = 0.0002
				39.2	p = 0.0014
				43.5	p = 0.0127
Figure S3K, left	Two-way RM ANOVA,	Time x Treatment: F (11, 286) = 2.789	p = 0.0018	Šídák correction	
	Time in Zone	Time: F (2.175, 56.55) = 32.24	p < 0.0001	for multiple comparisons	
		Treatment: F (1, 26) = 0.2474	p = 0.6231	SAL vs. HAL, significant temperature:	
		Subject: F (26, 286) = 8.030e-009	p > 0.9999	22.1	p = 0.0028

Figure S3K, right	Two-way RM ANOVA,	Time x Treatment: F (11, 286) = 5.082	p < 0.0001	Šídák correction	
	Zone entries	Time: F (2.603, 67.67) = 48.34	p < 0.0001	for multiple comparisons	
		Treatment: F (1, 26) = 24.04	p < 0.0001	SAL vs. HAL, significant temperature:	
		Subject: F (26, 286) = 3.108	p < 0.0001	17.8	p = 0.0280
				22.1	p = 0.0009
				26.4	p = 0.0085
Figure S3L, left	Two-way RM ANOVA,	Time x Treatment: F (11, 297) = 6.536	p < 0.0001	Šídák correction	
	Time in Zone	Time: F (2.754, 74.35) = 50.45	p < 0.0001	for multiple comparisons	
		Treatment: F (1, 27) = 2.646	p = 0.1155	SAL vs. COC, significant temperature:	
		Subject: F (27, 297) = 2.604e-008	p > 0.9999	9.3	p = 0.0297
				30.6	p = 0.0386
				43.5	p = 0.0002
				47.7	p = 0.0006
				52	p = 0.0007
Figure S3L, right	Two-way RM ANOVA,	Time x Treatment: F (11, 297) = 5.768	p < 0.0001	Šídák correction	
	Zone entries	Time: F (3.221, 86.98) = 54.82	p < 0.0001	for multiple comparisons	
		Treatment: F (1, 27) = 15.84	p = 0.0005	SAL vs. COC, significant temperature:	
		Subject: F (27, 297) = 61.99	p < 0.0001	22.1	p = 0.0369
				26.4	p = 0.01
				30.6	p = 0.0029
				34.9	p = 0.0027
				39.2	p = 0.0027
				43.5	p = 0.0047
				47.7	p = 0.0111
				52	p = 0.013
Figure S3M, left	Two-way RM ANOVA,	Time x Treatment : F (11, 286) = 1.945	p = 0.0338	Šídák correction	
	Time in Zone	Time: F (2.216, 57.62) = 46.14	p < 0.0001	for multiple comparisons	
		Treatment: F (1, 26) = 0.2727	p = 0.6059	SAL vs. CBD, significant temperature:	All ns
		Subject: F (26, 286) = 1.128e-008	p > 0.9999		
Figure S3M, right	Two-way RM ANOVA,	Time x Treatment: F (11, 286) = 0.7298	p = 0.7096		
	Zone entries	Time: F (1, 26) = 0.3000	p < 0.0001		
		Treatment: F (1, 26) = 3.663	p = 0.5886		
		Subject: F (26, 286) = 4.904	p < 0.0001		
Figure S5A, left	Two-way RM ANOVA,	Time x Treatment: F (59, 1534) = 1.415	p = 0.0222	Šídák correction	
	Temperature Preference	Time: F (9.121, 237.1) = 5.426	p < 0.0001	for multiple comparisons	
		Treatment: F (1, 26) = 5.820	p = 0.0232	SAL vs. MIH, significant minutes:	All ns

		Subject: F (26, 1534) = 37.62	p < 0.0001		
Figure S5A, right	Mixed-effects RM<br>Speed	Time x Treatment: F (59, 1528) = 1.244<br>Time: F (4.759, 123.3) = 1.621<br>Treatment: F (1, 26) = 0.3256	p = 0.1040<br>p = 0.1624<br>*p =0.5731*		
Figure S5B, left	Two-way RM ANOVA,<br>Temperature Preference	Time x Treatment: F (59, 1534) = 0.7614<br>Time: F (9.279, 241.2) = 4.765<br>Treatment: F (1, 26) = 1.722<br>Subject: F (26, 1534) = 56.87	p = 0.9090<br>p < 0.0001<br>p = 0.2009<br>p < 0.0001		
Figure S5B, right	Mixed-effects RM<br>Speed	Time x Treatment: F (59, 1534) = 0.9454<br>Time: F (14.07, 365.7) = 1.670<br>Treatment: F (1, 26) = 4.748	p = 0.5943<br>p = 0.0593<br>*p =0.0386*		
Figure S5C, left	Two-way RM ANOVA,<br>Temperature Preference	Time x Treatment: F (59, 1534) = 1.682<br>Time: F (6.451, 167.7) = 1.867<br>Treatment: F (1, 26) = 6.556<br>Subject: F (26, 1534) = 40.26	p = 0.0011<br>p = 0.0839<br>p =0.0166<br>p < 0.0001	Šídák correction<br>for multiple comparisons<br>SAL vs. 2- BFI, significant minutes:	All ns
Figure S5C, right	Mixed-effects RM<br>Speed	Time x Treatment: F (59, 1531) = 0.8806<br>Time: F (11.00, 285.6) = 1.940<br>Treatment: F (1, 26) = 16.29	p = 0.7277<br>p = 0.0344<br>*p =0.0004*		
Figure S5D, left	Two-way RM ANOVA,<br>Temperature Preference	Time x Treatment: F (59, 1593) = 1.745<br>Time: F (9.291, 250.8) = 3.448<br>Treatment: F (1, 27) = 0.001466<br>Subject: F (27, 1593) = 32.40	p =0.0005<br>p = 0.0004<br>p = 0.9697<br>p < 0.0001	Šídák correction<br>for multiple comparisons<br>SAL vs. 2- COC, significant minutes:	All ns
Figure S5D, right	Mixed-effects RM<br>Speed	Time x Treatment: F (59, 1593) = 2.024<br>Time: F (12.81, 345.9) = 2.741<br>Treatment: F (1, 27) = 37.37	p = 0.0011<br>p < 0.0001<br>p < 0.0001	Šídák correction<br>for multiple comparisons<br>SAL vs. 2- COC, significant minutes:	
				1	p < 0.0001
				2	p < 0.0001
				3	p = 0.0003
				4	p = 0.0054
				5	p < 0.0001
				6	p < 0.0001
				7	p < 0.0001
				8	p < 0.0001
				9	p < 0.0001

				10	p < 0.0001
				11	p = 0.0002
				12	p = 0.0002
				13	p < 0.0001
				14	p < 0.0001
				15	p = 0.0013
				16	p < 0.0001
				17	p = 0.0013
				18	p = 0.0004
				20	p < 0.0001
				21	p = 0.0014
				22	p = 0.0042
				23	p = 0.0056
				24	p = 0.0038
				27	p = 0.0083
				28	p = 0.0162
				41	p = 0.0101
				48	p = 0.0241
				52	p = 0.0007
				59	p = 0.0406
Figure S5E, left	Two-way RM ANOVA,	Time x Treatment: F (59, 1534) = 1.410	p = 0.0234	Šídák correction	
	Temperature Preference	Time: F (7.994, 207.8) = 6.000	p < 0.0001	for multiple comparisons	
		Treatment: F (1, 26) = 2.319	p = 0.1399	SAL vs. CBD, significant minutes:	All ns
		Subject: F (26, 1534) = 51.57	p < 0.0001		
Figure S5E, right	Mixed-effects RM	Time x Treatment: F (59, 1531) = 1.342	p = 0.0446	Šídák correction	
	Speed	Time: F (13.27, 344.3) = 2.315	p = 0.0056	for multiple comparisons	
		Treatment: F (1, 26) = 0.1219	*p =0.7298*	SAL vs. CBD, significant minutes:	All ns
Figure S7A	Unpaired t-test				
	Temperature Preference by sex				
	VEH	t(10) = 0.2593	p = 0.8007		
	TRV 1	t(10) = 0.5934	p = 0.5661		
	TRV 3	t(10) = 0.5713	p = 0.5804		
	TRV 10	t(10) = 0.5163	p = 0.6169		
	TRV 30	t(10) =1.011	p = 0.3358		
Figure S7B	Unpaired t-test				
	Speed by sex				
	VEH	t(10) = 1.237	p = 0.2443		

	TRV 1	t(10) = 0.4068	p = 0.2081		
	TRV 3	t(10) = 0.5713	p = 0.0641		
	TRV 10	t(10) = 1.397	p = 0.1927		
	TRV 30	t(10) =0.3480	p = 0.7351		
